# Lymphedema symptom trajectories and patient-reported outcomes after axillary lymph node dissection for breast cancer: A prospective cohort study

**DOI:** 10.1016/j.apjon.2026.101041

**Published:** 2026-10-05

**Authors:** Cuiju Wen, Hongjuan Sun, Xiang Li, Jingyan Xu, Jie Zhang, Lize Wang, Yang Yang, Zhaoqing Fan, Xinying Yu

**Affiliations:** Key Laboratory of Carcinogenesis and Translational Research (Ministry of Education), Breast Center, Peking University Cancer Hospital & Institute, Beijing, China

**Keywords:** Axillary lymph node dissection, Breast cancer-related lymphedema, Patient-reported outcomes, Symptom trajectories

## Abstract

**Objective:**

To characterize lymphedema symptom trajectories and associations with quality of life, anxiety, depression, and upper limb function in Chinese breast cancer survivors after axillary lymph node dissection (ALND).

**Methods:**

In this prospective cohort study, 332 women undergoing ALND at a cancer center were assessed preoperatively and annually through 24 months using the Breast Cancer and Lymphedema Symptom Experience Index (BCLE-SEI), Short Form-36 version 2 (SF-36v2; physical component summary [PCS], mental component summary [MCS]), Hospital Anxiety and Depression Scale (HADS), and Disabilities of the Arm, Shoulder, and Hand questionnaire (DASH). Group-based trajectory modeling (GBTM) identified trajectories; mixed-effects models compared patient-reported outcomes (PROs) changes across groups, and multivariable regression examined trajectory–PRO associations.

**Results:**

GBTM identified three trajectories: persistent-low (64.2%), high-stable (19.3%), and low-rapid-progression (16.6%). Significant group-by-time interactions emerged for PCS and DASH, with 12-month interactions for MCS and HADS subscales in the low-rapid-progression group. This group showed less PCS improvement and greater DASH deterioration at 12 and 24 months. For MCS, HADS-anxiety subscale (HADS-A), and HADS-depression subscale (HADS-D), interactions were significant at 12 but not 24 months, though outcomes remained worse in this group (physical–psychological dissociation). Low-rapid-progression membership was independently associated with poorer quality of life, greater upper limb impairment, and higher anxiety and depression risk (all *P* < 0.05).

**Conclusions:**

Symptom trajectories were independently associated with PROs after ALND. The low-rapid-progression trajectory was associated with poorer quality of life, greater upper limb impairment, and elevated anxiety and depression risk. Early identification may support risk-stratified surveillance, though whether targeted intervention improves PROs requires prospective studies.

**Trial registration:**

The study was registered in the Chinese Clinical Trial Registry (ChiCTR2200060685).

## Introduction

Although axillary surgery for breast cancer has been substantially de-escalated, axillary lymph node dissection (ALND) remains necessary for many patients with advanced nodal disease.^1^ In China, more than one-third of hospitalized patients with breast cancer present with axillary lymph node metastasis.^2^ ALND frequently causes upper-limb morbidity, including pain, limited mobility, reduced strength, and lymphedema,^3^ which affects up to 20% of patients.^4^ Breast cancer-related lymphedema (BCRL) is a debilitating treatment sequela characterized by abnormal lymph accumulation in the arm, shoulder, breast, or thoracic region. Given its clinical burden and multidimensional health effects, BCRL warrants prioritized evaluation and management.

Objective methods, including circumferential measurement, infrared perometry, and bioimpedance spectroscopy, are widely used but may not capture the dynamic course of lymphedema and can show substantial interoperator variability.^5^ Symptom assessment provides complementary information. First, hallmark symptoms such as heaviness, tightness, aching, and fullness are inherently subjective and can be captured only through patient self-report.^6^ Second, self-reported symptoms can predict lymphedema onset and may precede clinically detectable swelling, supporting earlier detection and intervention.^7^ Third, patient reports capture functional and psychosocial effects, such as interference with daily activities and emotional distress, that volumetric measurements cannot describe.^8^ Fourth, objective and subjective measures assess distinct constructs and correlate only modestly, so symptom assessment provides information unavailable from objective measurement alone.^9^^,^^10^ The International Society of Lymphology (ISL) Consensus Document defines Stage 0 lymphedema as symptomatic changes without clinically detectable edema,^11^ supporting symptom assessment before objective signs emerge. Although lymphedema stage is based on clinical findings such as pitting, reversibility with limb elevation, and tissue fibrosis rather than patient report, stage generally corresponds to more numerous and severe patient-experienced symptoms.^12^

A patient-reported outcome (PRO) is any aspect of health status reported directly by the patient without interpretation by a clinician or another person.^13^ Validated PRO instruments commonly assess symptom burden, mood, physical function, quality of life (QoL), and distress.^14^, ^15^, ^16^ PROs are widely used in cancer symptom management, clinical trials of antineoplastic agents, and evaluations of new therapeutic approaches.^15^^,^^17^^,^^18^ Because this study focused on symptom trajectories and their associations with PROs, the Breast Cancer and Lymphedema Symptom Experience Index (BCLE-SEI) was the primary symptom measure, with objective staging performed concurrently to characterize disease severity. Longitudinal evidence indicates that lymphedema-related impairment in health-related QoL can persist for up to 10 years after treatment, and cross-sectional studies associate lymphedema with poorer QoL and sleep.^19^^,^^20^ Previous trajectory studies have examined limb volume^21^ or combined biomarker and symptom patterns,^22^ but have not determined how distinct symptom trajectories relate to longitudinal changes in QoL, anxiety, depression, and upper-limb function. Evaluating trajectories rather than lymphedema presence or a single total symptom score may identify clinically meaningful subgroups with divergent courses, including patients whose initially mild symptoms progress rapidly after surgery. This prospective cohort study therefore characterized lymphedema symptom trajectories and their associations with the longitudinal course of PROs among breast cancer survivors after ALND.

## Methods

### Study procedures

Between June 2022 and July 2024, consecutive patients were prospectively enrolled in a BCRL early-detection cohort at Peking University Cancer Hospital. Eligible participants were women aged ≥ 18 years with histologically confirmed invasive breast cancer who underwent breast surgery with unilateral ALND and were willing and able to provide written informed consent. Exclusion criteria were bilateral breast cancer or bilateral ALND; previous ipsilateral breast surgery or ALND; pre-existing upper limb lymphedema of any cause; distant metastasis at diagnosis; cognitive impairment or psychiatric illness that precluded questionnaire completion; unavailable preoperative arm circumference measurements; or inability to attend postoperative follow-up at the study center. Research nurses recruited participants in the inpatient ward 1–2 days before surgery. Trained research nurses who were also certified lymphedema therapists conducted structured assessments at baseline in a private consultation room on the ward and at follow-up in the outpatient clinic. Each assessment lasted approximately 10 minutes and included the BCLE-SEI, Short Form-36 version 2 (SF-36v2), Hospital Anxiety and Depression Scale (HADS), and Disabilities of the Arm, Shoulder, and Hand questionnaire (DASH) questionnaires, objective arm circumference measurement, and clinical evaluation. Because the questionnaires contained more than 100 items, the research nurse assisted older adults or participants with lower educational level by reading response options aloud and clarifying items. Participants in whom clinically significant swelling was detected at any assessment were referred to certified lymphedema therapists as part of routine care. No serious adverse events occurred during follow-up.

The Research Ethics Committee of Peking University Cancer Hospital approved the study (Approval No. 2022KT78), which was registered in the Chinese Clinical Trial Registry (ChiCTR2200060685) and reported in accordance with the Strengthening the Reporting of Observational Studies in Epidemiology (STROBE) guideline.^23^ After ethics approval, the planned follow-up was extended from 12 to 60 months. The present analysis includes data through 24 months. All participants provided written informed consent before enrollment. The cohort size was determined a priori for the parent BCRL early-detection study to estimate the incidence of chronic clinical lymphedema under prospective surveillance. This symptom-trajectory study was a secondary analysis with no separate a priori power calculation. Reasons for missing data and comparisons between participants who completed follow-up and those who did not are reported in the Results and Supplementary Table S1.

### Measurements

#### Clinical and demographic characteristics

At baseline, structured interviews collected demographic data, including date of birth, educational level, marital status, employment status, height, weight, medical insurance status, smoking history, and hand dominance. Medical record review provided clinical and treatment information, including clinical and pathological tumor-node-metastasis stage, comorbidities, Eastern Cooperative Oncology Group performance status (ECOG PS), surgical procedures, chemotherapy, radiotherapy, and endocrine therapy.

#### Lymphedema symptoms

Lymphedema symptoms were assessed using Part I of the BCLE-SEI. This validated, lymphedema-specific instrument assesses symptom occurrence in Part I and symptom-related distress in Part II.^24^ Part I comprises 24 symptoms: limited movement of the shoulder, elbow, wrist, fingers, and arm; swelling of the arm, breast, and chest wall; stiffness; tightness; heaviness; fibrosis, defined as skin toughness or thickening; firmness; tenderness; hotness; redness; blistering; pain, aching, or soreness; numbness; burning; stabbing; tingling; fatigue; and weakness. Each symptom is rated on a five-point Likert scale, from 0 (none) to 4 (severe). Total scores range from 0 to 96, with higher scores indicating more severe symptoms. The Chinese version of the BCLE-SEI is validated in Chinese populations.^25^

#### Objective lymphedema assessment

Objective lymphedema was assessed using serial arm circumference measurements with an inelastic tape at the ulnar styloid and at 4-cm intervals proximally to the axilla. Relative volume change (RVC) was calculated, and lymphedema was defined as an RVC ≥ 10% from baseline.

#### Quality of life

Health-related QoL was assessed using the SF-36v2. Its 36 items assess eight domains: Physical Functioning, Role-Physical, Bodily Pain, General Health, Vitality, Social Functioning, Role-Emotional, and Mental Health. Domain scores are used to derive the Physical Component Summary (PCS) and Mental Component Summary (MCS) scores. Scores range from 0 to 100, with higher scores indicating better health-related QoL. Following the norm-based scoring protocol, PCS, MCS, and domain scores are standardized to a mean of 50 and a standard deviation (SD) of 10 for the Chinese reference population. The Chinese SF-36v2 has robust psychometric properties.^26^

#### Anxiety and depression

Anxiety and depression were assessed using the HADS. The 14-item instrument contains two 7-item subscales assessing anxiety (HADS-A) and depression (HADS-D). Each item is scored from 0 to 3, yielding subscale scores of 0–21, with higher scores indicating greater symptom severity. Scores of 0–7 indicate no clinically relevant symptoms, 8–10 borderline symptoms, and 11–21 clinically significant symptoms. For the analyses, HADS-A and HADS-D were dichotomized at a score ≥ 8, indicating possible anxiety or depression symptoms. The Chinese HADS has strong psychometric validity among patients with cancer.^27^

#### Upper limb function

Upper limb function was assessed using the DASH, developed in 1996 through collaboration among the American Academy of Orthopedic Surgeons, the Council of Musculoskeletal Specialty Societies, and the Institute for Work and Health.^28^ This study used the 30-item disability and symptom component. Items are scored from 1 to 5. The total score is the mean item score minus 1, multiplied by 25, producing a score from 0 to 100. Higher scores indicate greater upper limb disability. The Chinese DASH has satisfactory reliability and validity.^29^

Lymphedema symptoms and PROs were assessed preoperatively and at 12 and 24 months after ALND. The same specialist nurse conducted each participant's preoperative and postoperative assessments. Two reviewers independently entered questionnaire data and reconciled discrepancies.

### Statistical analysis

Baseline demographic and clinical characteristics were summarized for the full cohort and compared across lymphedema symptom trajectory groups. Continuous variables are presented as means with SDs or medians with interquartile ranges. Categorical variables are presented as counts and percentages. Continuous variables were compared using analysis of variance or the Kruskal–Wallis test according to distributional assumptions. Categorical variables were compared using the chi-square or Fisher exact test when expected cell counts were small.

Group-based trajectory modeling (GBTM) identified distinct longitudinal patterns of lymphedema symptoms from baseline through postoperative follow-up. Model selection considered the Bayesian information criterion, Akaike information criterion, sample-size-adjusted Bayesian information criterion, and entropy. Additional criteria required an average posterior probability of group assignment ≥ 0.7 and > 5% of participants in each trajectory group. In simple terms, GBTM groups patients according to the pattern of their symptom change over time (e.g., whether symptoms remain low, stay high, or rise sharply after surgery), rather than relying on a single symptom measurement.

PROs were compared across trajectory groups using mixed-effects models for repeated measures (MMRM). Fixed effects included trajectory group, assessment time (baseline, 12, and 24 months), weight change from baseline as a time-varying covariate, and the group-by-time interaction. HADS-A and HADS-D were modeled as binary outcomes (score ≥ 8) using generalized linear mixed-effects models with a logit link, fitted by adaptive Gauss–Hermite quadrature, with results expressed as odds ratios. A participant-specific random intercept accounted for within-participant correlation. The interaction tested whether PRO changes differed across trajectory groups. PROs were modeled as outcomes rather than time-varying covariates. Thus, GBTM was used first to derive the symptom-trajectory groups from the data, and the mixed-effects models were then used to test whether these groups differed in PROs over time; the two methods therefore serve sequential, complementary roles rather than addressing overlapping questions.

Regression models evaluated associations between symptom-trajectory membership and PROs. Linear regression was used for continuous PCS, MCS, and DASH scores, reported as regression coefficients (*β*) and 95% confidence intervals (CIs). Logistic regression was used for the binary HADS-A and HADS-D outcomes (score  ≥  8), reported as odds ratios and 95% CIs. Covariates were selected a priori for clinical relevance and published evidence, irrespective of univariable significance; univariable associations are reported in Supplementary Table S2. Three hierarchical models were fitted. Model 1 adjusted for age, educational level, marital status, employment status, body mass index (BMI), and follow-up time (12 vs. 24 months). Model 2 additionally adjusted for whether ALND affected the dominant arm, chemotherapy, taxane-based chemotherapy, and radiotherapy. Model 3 additionally adjusted for baseline SF-36v2 PCS, MCS, HADS, and DASH scores, and for weight change from baseline at the same follow-up assessment as the outcome. ECOG PS, smoking history, and medical insurance status were not included because their minimal variability precluded stable estimation: all participants had an ECOG PS of 0, 3 (0.9%) reported ever smoking, and 9 (2.7%) had government-funded medical insurance. Trends across trajectory groups were also tested. Primary regression models used conventional standard errors; sensitivity analyses used Huber–White robust standard errors to assess heteroscedasticity (Supplementary Table S3). Each model used the participant's last available follow-up outcome, whereas MMRM addressed the longitudinal repeated-measures structure. Baseline and follow-up BCLE-SEI symptom severity were examined in separate models rather than simultaneously with trajectory membership to avoid overadjustment and collinearity.

Stratified analyses evaluated age (< 50 vs. ≥ 50 years), educational level (< 12 vs. ≥ 12 years), marital status (partnered vs. single), employment status (employed vs. not employed), BMI (< 25 vs. ≥ 25 kg/m^2^), whether ALND affected the dominant arm, taxane-based chemotherapy, and radiotherapy. Likelihood-ratio tests assessed interactions. The 50-year threshold was selected for consistency with previous studies in this population.^30^ In exploratory subgroup analyses, HADS-A and HADS-D were analyzed as continuous scores from 0 to 21 rather than as binary outcomes to preserve statistical power because few participants within subgroups had scores ≥ 8.

The frequency and pattern of missing data by assessment and instrument are reported in Supplementary Table S1. GBTM used full-information maximum likelihood estimation, incorporating all available longitudinal observations. For the continuous outcomes, mixed-effects models for repeated measures were fitted by restricted maximum likelihood; for the binary HADS outcomes, generalized linear mixed-effects models with a logit link were fitted by adaptive Gauss–Hermite quadrature. Both approaches accommodated unbalanced observations across time. Two sensitivity analyses assessed robustness. First, regression models were refitted among participants with complete outcome data at all three assessments, using the 24-month outcome, to assess whether differential attrition influenced the results (Supplementary Table S4). Second, to assess whether functional symptom items overlapping with DASH and SF-36v2 physical domains inflated the associations, trajectories were re-derived using only the 12 swelling- or edema-specific BCLE-SEI items: arm, breast, and chest-wall swelling; stiffness; tightness; heaviness; fibrosis; firmness; tenderness; hotness; redness; and blistering. Regression models were then repeated using these trajectories as the exposure (Supplementary Table S5). E-values were calculated for the low-rapid-progression group versus the persistent-low group to assess robustness to unmeasured confounding.^31^ The E-value represents the minimum association, on the risk-ratio scale, that an unmeasured confounder would need to have with both trajectory membership and the outcome, conditional on measured covariates, to explain the observed association fully (Supplementary Table S6).

Analyses were performed using *R* version 4.2.2 (*R* Foundation for Statistical Computing) and the Free Statistics analysis platform (Beijing, China). A two-sided *P* value < 0.05 indicated statistical significance.

## Results

### Clinical and demographic characteristics of the participants

This study included 332 breast cancer survivors following unilateral ALND, with the patient selection process detailed in Fig. 1. The mean age was 47.6 (9.4) years (Table 1). The median follow-up duration was 12.0 (12.0, 23.0) months post-surgery. Of the 332 participants, 332 (100.0%), 331 (99.7%), and 118 (35.5%) completed the baseline, 12-month, and 24-month assessments, respectively; 117 (35.2%) had data at all three assessments (Supplementary Table S1). Missing 24-month data were primarily attributable to staggered enrollment (*n* = 147), with a further 67 participants not returning after the 12-month visit because their follow-up had been transferred to other hospitals (Supplementary Table S1).

**Fig. 1:**
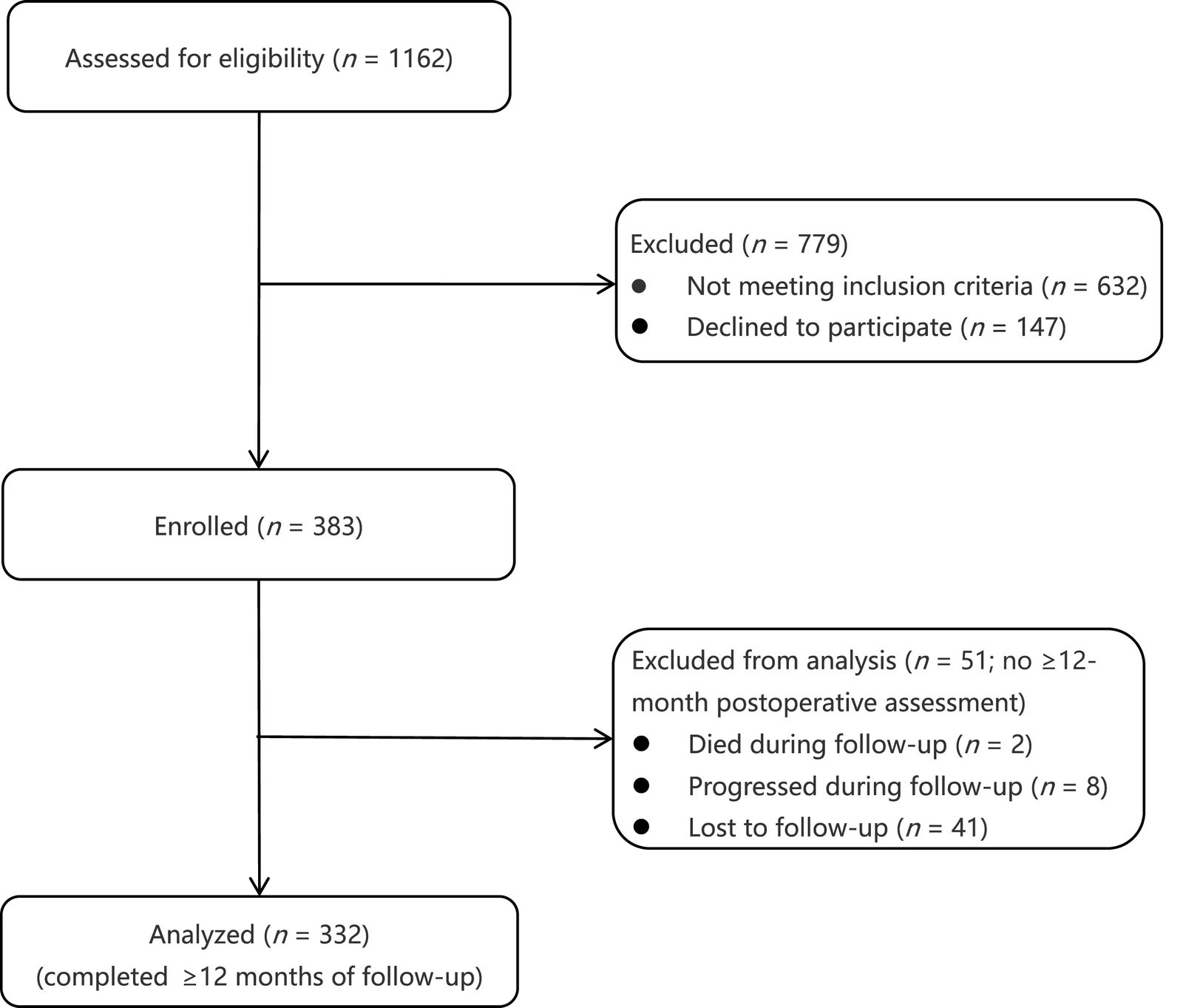
Flowchart of participant selection.

**Table 1:** Demographic and clinical characteristics of the study cohort, stratified by lymphedema symptom trajectory group.

Variables	Total (*n* = 332)	Lymphedema symptom trajectory group			*P* value
		Persistent-low (*n* = 213)	High-stable (*n* = 64)	Low-rapid-progression (*n* = 55)	
Age (years), Mean (SD)	47.6 (9.4)	48.0 (9.7)	46.7 (8.7)	46.9 (9.2)	0.548
Educational level (years), *n* (%)					0.176
< 12	129 (38.9)	90 (42.3)	23 (35.9)	16 (29.1)	
≥ 12	203 (61.1)	123 (57.7)	41 (64.1)	39 (70.9)	
Marital status, *n* (%)					0.338
Married or living with a partner	292 (88.0)	189 (88.7)	53 (82.8)	50 (90.9)	
Living alone	40 (12.0)	24 (11.3)	11 (17.2)	5 (9.1)	
Employment status, *n* (%)					0.285
Employed	194 (58.4)	118 (55.4)	37 (57.8)	39 (70.9)	
Unemployed	47 (14.2)	31 (14.6)	11 (17.2)	5 (9.1)	
Retired	91 (27.4)	64 (30.0)	16 (25.0)	11 (20.0)	
BMI (kg/m^2^), Mean (SD)	24.6 (3.6)	24.6 (3.7)	25.0 (3.8)	24.5 (3.4)	0.664
Medical insurance status, *n* (%)					0.295
Basic medical insurance	323 (97.3)	205 (96.2)	64 (100.0)	54 (98.2)	
Government-funded medical insurance	9 (2.7)	8 (3.8)	0 (0.0)	1 (1.8)	
Smoking history, *n* (%)					1.000
Never	329 (99.1)	210 (98.6)	64 (100.0)	55 (100.0)	
Ever	3 (0.9)	3 (1.4)	0 (0.0)	0 (0.0)	
ALND affected dominant arm, *n* (%)					0.200
None	172 (51.8)	117 (54.9)	27 (42.2)	28 (50.9)	
Yes	160 (48.2)	96 (45.1)	37 (57.8)	27 (49.1)	
Affected side, *n* (%)					0.738
Left	175 (52.7)	115 (54.0)	31 (48.4)	29 (52.7)	
Right	157 (47.3)	98 (46.0)	33 (51.6)	26 (47.3)	
cTNM stage, *n* (%)					0.407
I	3 (0.9)	1 (0.5)	1 (1.6)	1 (1.8)	
II	277 (83.4)	178 (83.6)	51 (79.7)	48 (87.3)	
III	52 (15.7)	34 (16.0)	12 (18.8)	6 (10.9)	
pTNM stage, *n* (%)					0.538
0	77 (23.2)	54 (25.4)	13 (20.3)	10 (18.2)	
I	32 (9.6)	22 (10.3)	6 (9.4)	4 (7.3)	
II	142 (42.8)	85 (39.9)	33 (51.6)	24 (43.6)	
III	81 (24.4)	52 (24.4)	12 (18.8)	17 (30.9)	
CCI, Median (IQR)	1.0 (0.0, 2.0)	1.0 (1.0, 2.0)	1.0 (0.8, 2.0)	1.0 (0.0, 2.0)	0.490
ECOG PS, *n* (%)					–
0	332 (100.0)	213 (100.0)	64 (100.0)	55 (100.0)	
Breast surgery, *n* (%)					0.531
BCS	95 (28.6)	65 (30.5)	15 (23.4)	15 (27.3)	
Mastectomy	237 (71.4)	148 (69.5)	49 (76.6)	40 (72.7)	
No. of nodes removed, Mean (SD)	21.3 (8.2)	21.3 (7.3)	20.0 (7.3)	22.5 (11.7)	0.259
No. of positive nodes, Median (IQR)	1.0 (0.0, 3.0)	1.0 (0.0, 3.0)	1.0 (0.0, 2.2)	1.0 (0.0, 4.0)	0.547
Chemotherapy, *n* (%)					0.518
None	32 (9.6)	23 (10.8)	4 (6.2)	5 (9.1)	
Neoadjuvant chemotherapy	220 (66.3)	141 (66.2)	40 (62.5)	39 (70.9)	
Adjuvant chemotherapy	80 (24.1)	49 (23.0)	20 (31.2)	11 (20.0)	
Taxane-based chemotherapy, *n* (%)					0.138
None	68 (20.5)	49 (23.0)	9 (14.1)	10 (18.2)	
Neoadjuvant	183 (55.1)	118 (55.4)	32 (50.0)	33 (60.0)	
Adjuvant	81 (24.4)	46 (21.6)	23 (35.9)	12 (21.8)	
Radiotherapy, *n* (%)					0.489
None	61 (18.4)	42 (19.7)	12 (18.8)	7 (12.7)	
Yes	271 (81.6)	171 (80.3)	52 (81.2)	48 (87.3)	
Endocrine therapy, *n* (%)					0.767
None	88 (26.5)	53 (24.9)	18 (28.1)	17 (30.9)	
TAM	38 (11.4)	27 (12.7)	7 (10.9)	4 (7.3)	
AI	206 (62.0)	133 (62.4)	39 (60.9)	34 (61.8)	
Lymphedema (RVC ≥ 10%)					0.903
No	301 (90.7)	194 (91.1)	58 (90.6)	49 (89.1)	
Yes	31 (9.3)	19 (8.9)	6 (9.4)	6 (10.9)	
Baseline PCS, Mean (SD)	46.8 (11.0)	49.4 (9.9)	39.5 (11.3)	45.4 (11.0)	< 0.001
Baseline MCS, Mean (SD)	43.3 (15.2)	45.7 (14.5)	37.8 (15.8)	40.9 (15.3)	< 0.001
Baseline DASH, Median (IQR)	0.8 (0.0, 7.7)	0.0 (0.0, 3.3)	11.2 (2.3, 21.0)	2.5 (0.0, 9.2)	< 0.001
Baseline HADS-A, *n* (%)					0.004
< 8	245 (73.8)	170 (79.8)	40 (62.5)	35 (63.6)	
≥ 8	87 (26.2)	43 (20.2)	24 (37.5)	20 (36.4)	
Baseline HADS-D, *n* (%)					0.002
< 8	277 (83.4)	188 (88.3)	45 (70.3)	44 (80.0)	
≥ 8	55 (16.6)	25 (11.7)	19 (29.7)	11 (20.0)	
Follow-up PCS, Mean (SD)	45.6 (9.8)	48.3 (8.3)	43.0 (9.5)	37.8 (10.8)	< 0.001
Follow-up MCS, Mean (SD)	49.1 (13.0)	52.7 (10.4)	43.4 (14.3)	41.6 (14.9)	< 0.001
Follow-up DASH, Median (IQR)	9.2 (2.5, 20.0)	5.0 (0.8, 11.7)	17.9 (7.5, 27.5)	25.0 (16.7, 29.6)	< 0.001
Follow-up HADS-A, *n* (%)					< 0.001
< 8	281 (84.6)	196 (92.0)	49 (76.6)	36 (65.5)	
≥ 8	51 (15.4)	17 (8.0)	15 (23.4)	19 (34.5)	
Follow-up HADS-D, *n* (%)					< 0.001
< 8	291 (87.7)	201 (94.4)	48 (75.0)	42 (76.4)	
≥ 8	41 (12.3)	12 (5.6)	16 (25.0)	13 (23.6)	

SD, standard deviation; IQR, interquartile range; BMI, body mass index; cTNM, clinical tumor-node-metastasis stage; pTNM, pathological tumor-node-metastasis stage; CCI, Charlson Comorbidity Index; ECOG PS, Eastern Cooperative Oncology Group performance status; ALND, axillary lymph node dissection; BCS, breast-conserving surgery; TAM, tamoxifen; AI, aromatase inhibitor; RVC, relative volume change; PCS, Physical Component Summary; MCS, Mental Component Summary; DASH, Disabilities of the Arm, Shoulder, and Hand questionnaire; HADS-A, Hospital Anxiety and Depression Scale–anxiety subscale; HADS-D, Hospital Anxiety and Depression Scale–depression subscale.

Smoking history was categorized as never (no history of smoking) or ever (former or current smoker). All participants had an ECOG PS of 0; the *P*-value was not calculated because of the absence of variability.

Overall, 44% of participants reported subjective arm swelling at any follow-up assessment (Supplementary Table S7), whereas 9.3% met the objective criterion for lymphedema (RVC ≥ 10% from baseline) at any follow-up assessment (Table 1).

We employed GBTM to determine the optimal number of lymphedema symptom trajectories (Table 2). The BIC was lowest for the three-trajectory model. Although the AIC and SABIC favored a five-trajectory solution, that solution included an empty class and was therefore rejected. The three-trajectory model also satisfied the quality criteria of more than 5% of participants in each group and an average posterior probability ≥ 0.7, and was selected as optimal. Fig. 2 presents the three distinct trajectories: persistent-low (64.2%), high-stable (19.3%), and low-rapid-progression (16.6%). The trajectory names reflect the shape of each group's symptom course: persistent-low (symptom scores remaining low throughout), high-stable (elevated symptoms at baseline that persist without substantial escalation), and low-rapid-progression (low initial symptoms followed by sharp escalation after surgery).

**Table 2:** Fit statistics for the lymphedema symptom trajectory models.

Fit statistic	Number of classes			
	2	3	4	5
BIC^a^	3243.85	2616.52	2851.96	2640.28
AIC^a^	3186.77	2540.41	2756.84	2526.13
SABIC^a^	3196.27	2553.07	2772.66	2545.12
Entropy^b^	0.99	0.88	0.85	0.99
Class proportion^c^	Class 1, 78.0%	Class 1, 64.2%	Class 1, 10.8%	Class 1, 0.0%
	Class 2, 22.0%	Class 2, 19.3%	Class 2, 56.3%	Class 2, 15.7%
		Class 3, 16.6%	Class 3, 19.3%	Class 3, 78.6%
			Class 4, 13.6%	Class 4, 5.7%
				Class 5, 0.0%
PP^d^	Class 1, 0.99	Class 1, 0.95	Class 1, 0.76	Class 1, 0.00
	Class 2, 0.99	Class 2, 0.99	Class 2, 0.93	Class 2, 0.99
		Class 3, 0.92	Class 3, 0.99	Class 3, 0.99
			Class 4, 0.93	Class 4, 0.99
				Class 5, 0.00

AIC, Akaike^,^s information criterion; BIC, Bayesian information criterion; SABIC, sample-size-adjusted Bayesian information criterion; PP, posterior probability.

a Lower values indicate better model fit.

b Values range from 0 to 1; higher values indicate better model fit.

c Each class of the retained models comprises at least 5% of the total sample.

d Higher values indicate better classification (preferably > 0.7 per class).

**Fig. 2:**
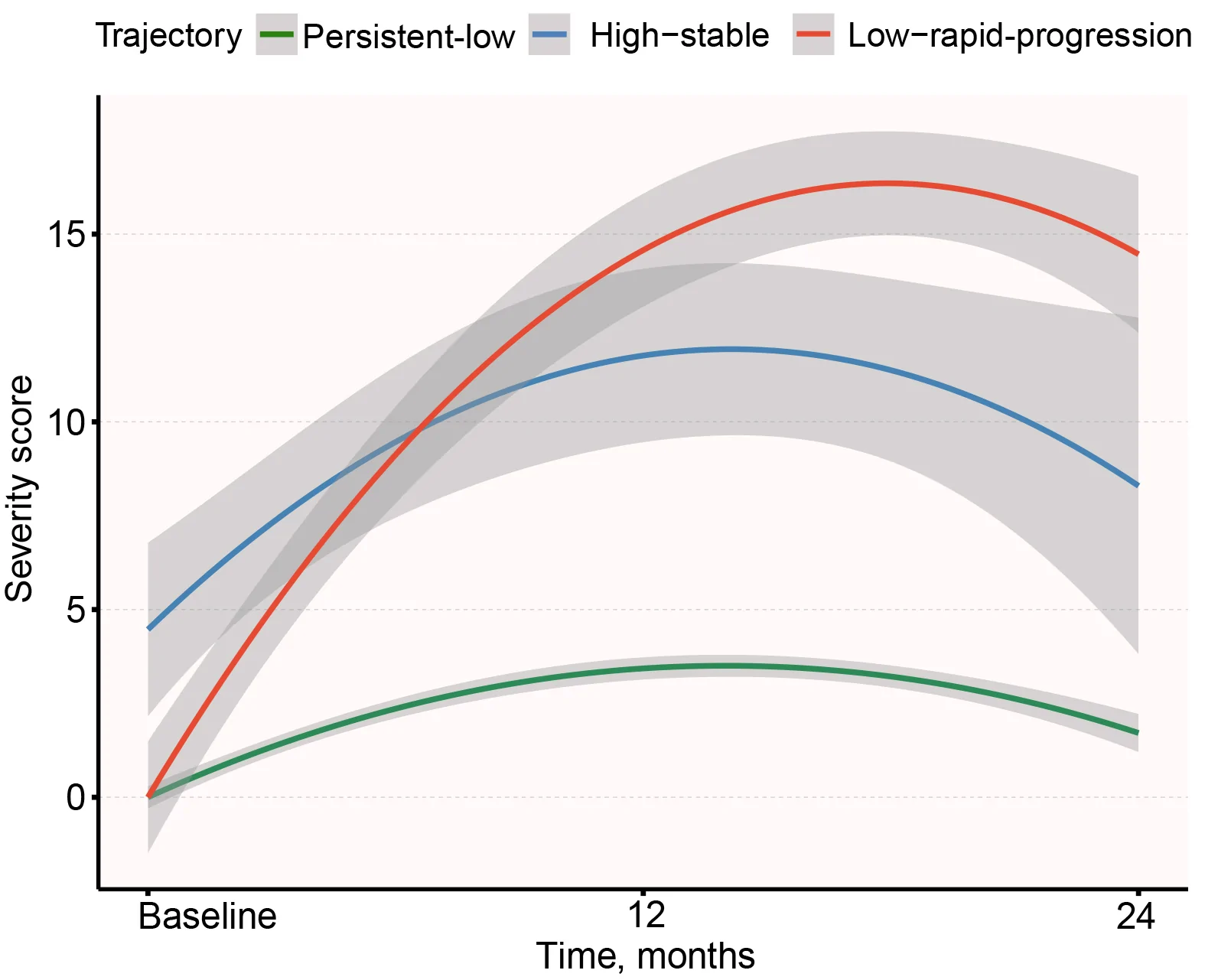
Lymphedema symptom trajectories during follow-up. Lines represent the mean BCLE-SEI symptom severity scores over time for each trajectory group. BCLE-SEI, Breast Cancer and Lymphedema Symptom Experience Index.

Baseline demographic and clinical characteristics stratified by lymphedema symptom trajectory group are summarized in Table 1. No baseline demographic, clinical, or treatment variables differed significantly across trajectory groups (all *P* > 0.05). All participants had an ECOG PS of 0, and only three (0.9%) reported a history of smoking; these variables therefore had negligible variability and could not differentiate the trajectory groups. By contrast, all baseline PRO measures (PCS, MCS, HADS, and DASH) showed highly significant between-group differences (all *P* < 0.01), as did all follow-up PRO assessments (all *P* < 0.01).

### Temporal patterns of PROs according to lymphedema symptom trajectories

#### Quality of life

PCS trajectories differed across groups (Fig. 3A, Supplementary Table S8). At baseline, PCS was significantly lower in the high-stable trajectory group (β = −9.92, 95% CI: −12.61 to −7.23; P < 0.001) and the low-rapid-progression trajectory group (β = −4.02, 95% CI: −6.88 to −1.16; P = 0.006) than in the persistent-low trajectory group. In the persistent-low group, PCS decreased significantly at 12 months (β = −1.64, 95% CI: −3.27 to −0.01; P = 0.049) but did not differ significantly from baseline at 24 months (β = 0.56, 95% CI: −1.82 to 2.94; P = 0.644). Group-by-time interactions indicated that the high-stable trajectory group showed a greater increase in PCS than the persistent-low trajectory group at 12 months (β = 4.45, 95% CI: 1.05 to 7.84; P = 0.011), whereas the difference at 24 months was not statistically significant (β = 4.61, 95% CI: −0.78 to 10.00; P = 0.094). Conversely, the low-rapid-progression trajectory group showed greater reductions in PCS than the persistent-low trajectory group at both 12 months (β = −5.61, 95% CI: −9.23 to −1.98; P = 0.003) and 24 months (β = −6.97, 95% CI: −11.64 to −2.31; P = 0.004).

**Fig. 3:**
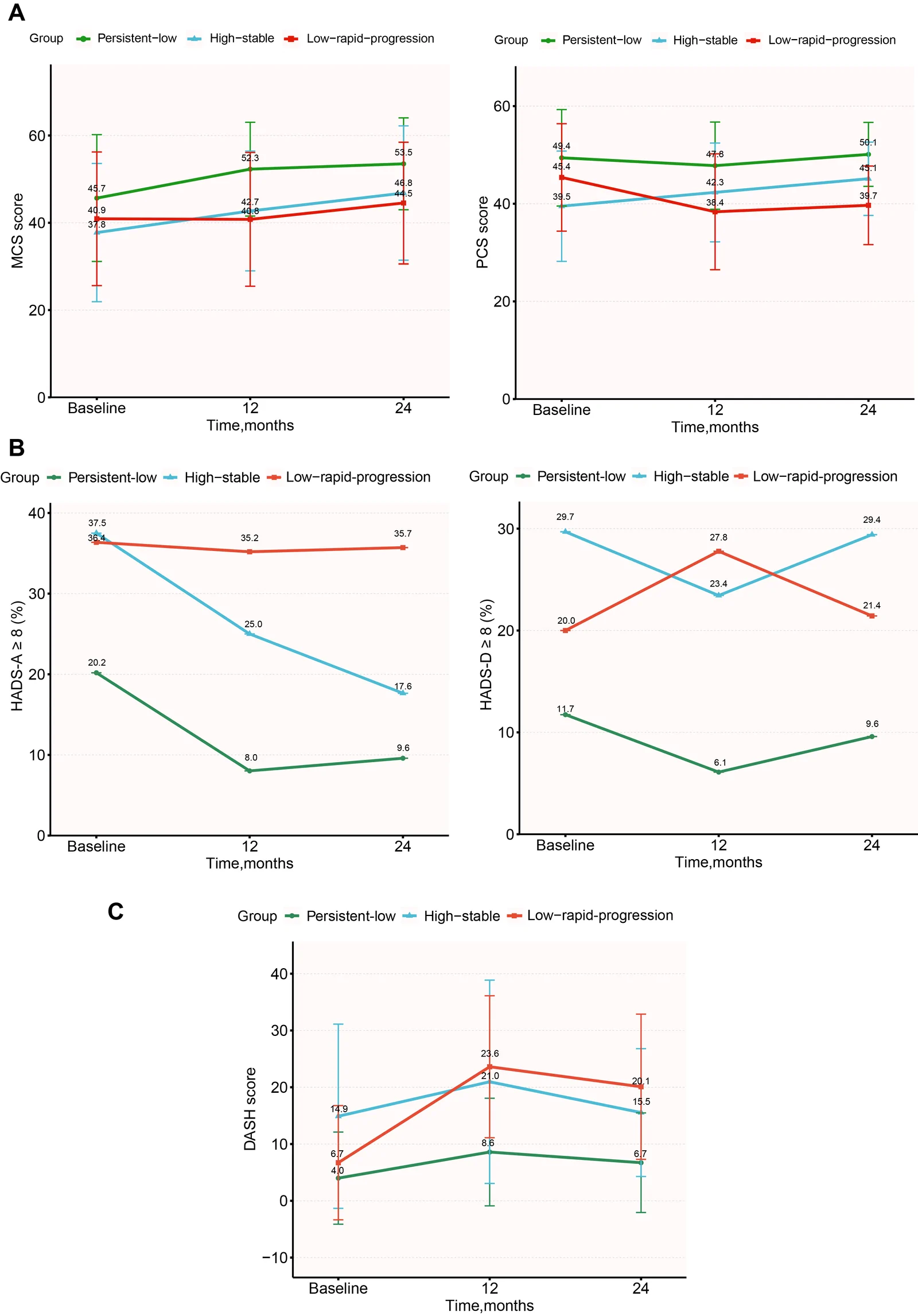
Time course of PROs stratified by lymphedema symptom trajectory group. (A) PCS and MCS scores; (B) proportion of participants with HADS-A or HADS-D score ≥ 8; (C) DASH scores. Associations are reported as regression coefficients (*β*) for continuous outcomes and odds ratios (OR) for binary HADS outcomes (score ≥ 8) (Supplementary Table S8). PROs, patient-reported outcomes; PCS, Physical Component Summary; MCS, Mental Component Summary; HADS-A, Hospital Anxiety and Depression Scale–anxiety subscale; HADS-D, Hospital Anxiety and Depression Scale–depression subscale; DASH, disabilities of the arm, shoulder, and hand questionnaire.

MCS improved in the persistent-low and high-stable groups, whereas the low-rapid-progression group showed no significant improvement at 12 months (Fig. 3A, Supplementary Table S8). At baseline, both the high-stable trajectory group (*β* = −7.90, 95% CI: −11.64 to −4.16; *P* < 0.001) and the low-rapid-progression trajectory group (*β* = −4.74, 95% CI: −8.71 to −0.78; *P* = 0.019) showed significantly lower MCS than the persistent-low trajectory group. Significant time effects were observed, with scores increasing by 6.63 units (95% CI: 4.68 to 8.58; *P* < 0.001) at 12 months and 8.28 units (95% CI: 5.36 to 11.20; *P* < 0.001) at 24 months relative to baseline. A significant group-by-time interaction emerged for the low-rapid-progression trajectory group at 12 months (*β* = −6.90, 95% CI: −11.23 to −2.56; *P* = 0.002), indicating attenuated improvement relative to the persistent-low trajectory group. No significant interactions were detected for the high-stable trajectory group at either time point (12 months: *P* = 0.416; 24 months: *P* = 0.739). For the low-rapid-progression trajectory group, the group-by-time interaction was no longer statistically significant at 24 months (*P* = 0.095), although the model-estimated MCS remained lower in this group than in the persistent-low group.

#### Anxiety and depression

HADS-A risk declined in the persistent-low and high-stable trajectory groups, whereas the low-rapid-progression group showed a less marked reduction in anxiety risk (Fig. 3B, Supplementary Table S8). At baseline, both the high-stable trajectory group (*OR* = 3.77, 95% CI: 1.45 to 9.82; *P* = 0.007) and the low-rapid-progression trajectory group (*OR* = 4.03, 95% CI: 1.46 to 11.13; *P* = 0.007) had significantly higher odds of HADS-A than the persistent-low trajectory group. Significant time effects were observed, with odds decreasing at 12 months (*OR* = 0.26, 95% CI: 0.13 to 0.52; *P* < 0.001) and 24 months (*OR* = 0.31, 95% CI: 0.11 to 0.87; *P* = 0.026) relative to baseline. A significant group-by-time interaction was detected for the low-rapid-progression trajectory group at 12 months (*OR* = 3.60, 95% CI: 1.07 to 12.10; *P* = 0.038), indicating attenuated reduction in odds relative to the persistent-low trajectory group. No significant interactions were observed for the high-stable trajectory group at either time point (12 months: *P* = 0.483; 24 months: *P* = 0.547) or for the low-rapid-progression trajectory group at 24 months (*P* = 0.213). Nevertheless, the model-estimated odds of HADS-A ≥ 8 remained higher in the low-rapid-progression group than in the persistent-low group at 24 months.

HADS-D risk declined in the persistent-low and high-stable groups at 12 months, whereas the low-rapid-progression group showed an increase in depression risk (Fig. 3B, Supplementary Table S8). At baseline, the high-stable trajectory group had substantially higher odds of the HADS-D than the persistent-low trajectory group (*OR* = 6.40, 95% CI: 1.78 to 23.00; *P* = 0.004), whereas the low-rapid-progression trajectory group did not differ significantly (*OR* = 2.39, 95% CI: 0.59 to 9.79; *P* = 0.225). A significant time effect was observed at 12 months (*OR* = 0.34, 95% CI: 0.13 to 0.84; *P* = 0.020) but not at 24 months (*OR* = 0.57, 95% CI: 0.18 to 1.83; *P* = 0.342). A significant group-by-time interaction was detected for the low-rapid-progression trajectory group at 12 months (*OR* = 7.69, 95% CI: 1.47 to 40.34; *P* = 0.016), indicating that the odds of HADS-D ≥8 increased in this group relative to the persistent-low trajectory group. No significant interactions were observed for the high-stable trajectory group at either time point (12 months: *P* = 0.474; 24 months: *P* = 0.900) or for the low-rapid-progression trajectory group at 24 months (*P* = 0.685). Nevertheless, the model-estimated odds of HADS-D ≥ 8 remained higher in the low-rapid-progression group than in the persistent-low group at 24 months.

#### Upper limb function

DASH scores worsened across all groups, with the low-rapid-progression group deteriorating most rapidly despite a baseline indistinguishable from the persistent-low group (Fig. 3C, Supplementary Table S8). At baseline, the high-stable trajectory group demonstrated significantly worse DASH scores than the persistent-low trajectory group (*β* = 10.90, 95% CI: 7.81 to 14.00; *P* < 0.001), whereas the low-rapid-progression trajectory group did not differ significantly (*β* = 2.72, 95% CI: −0.57 to 6.00; *P* = 0.105). Significant time effects were observed, with dysfunction scores increasing by 4.64 points (95% CI: 2.83 to 6.46; *P* < 0.001) at 12 months and 2.99 points (95% CI: 0.33 to 5.66; *P* = 0.028) at 24 months relative to baseline. Pronounced group-by-time interactions emerged for the low-rapid-progression trajectory group. This group exhibited significantly amplified deterioration at 12 months (*β* = 12.60, 95% CI: 8.57 to 16.63; *P* < 0.001), and this accelerated functional decline persisted at 24 months (*β* = 11.41, 95% CI: 6.19 to 16.62; *P* < 0.001). No significant interactions were detected for the high-stable trajectory group at either time point (12 months: *P* = 0.451; 24 months: *P* = 0.758).

### Associations between the lymphedema symptom trajectories and PROs

The regression analyses revealed significant associations between lymphedema symptom trajectories and PROs (Table 3). Compared with the persistent-low trajectory group, the low-rapid-progression trajectory group showed stronger negative associations with PCS and MCS scores (Model III: *β* = −9.99, 95% CI: −12.65 to −7.34, *P* < 0.001 for PCS; *β* = −9.53, 95% CI: −12.92 to −6.13, *P* < 0.001 for MCS) and greater functional impairment as reflected by higher DASH scores (Model III: *β* = 14.44, 95% CI: 10.98 to 17.89, *P* < 0.001). This trajectory group also showed increased odds of possible anxiety and depression symptoms (Model III: *OR* = 4.75, 95% CI: 1.96 to 11.48, *P* = 0.001 for HADS-A; *OR* = 4.21, 95% CI: 1.57 to 11.27, *P* = 0.004 for HADS-D). Follow-up lymphedema symptom severity consistently showed significant negative associations with PCS and MCS and positive associations with DASH scores (indicating worse function), HADS-A, and HADS-D across all models. In a sensitivity analysis restricted to the 117 participants with complete data at all three time points, all trajectory–PRO associations remained directionally consistent with the primary analysis (Supplementary Table S4). Re-deriving the trajectories from the 12 swelling- or edema-specific BCLE-SEI items yielded a three-trajectory solution with a composition similar to the primary analysis, and the trajectory–PRO associations remained directionally consistent and of similar magnitude across all outcomes, including DASH, supporting the robustness of the findings to potential measurement overlap (Supplementary Table S5).

**Table 3:** Associations between the lymphedema symptom trajectories and PROs.

Variable	PCS		MCS		DASH		HADS-A		HADS-D	
	*β* (95% CI)	*P* value	*β* (95% CI)	*P* value	*β* (95% CI)	*P* value	OR (95% CI)	*P* value	OR (95% CI)	*P* value
	Crude		Crude		Crude		Crude		Crude	
Trajectory groups										
Persistent-low	0 (Ref)		0 (Ref)		0 (Ref)		1 (Ref)		1 (Ref)	
High-stable	−5.28 (−7.80 to −2.76)	<0.001	−9.24 (−12.63 to −5.85)	<0.001	11.97 (8.70 to 15.25)	<0.001	3.53 (1.65 to 7.56)	0.001	5.58 (2.48 to 12.58)	<0.001
Low-rapid-progression	−10.47 (−13.14 to −7.80)	<0.001	−11.12 (−14.72 to −7.52)	< 0.001	15.31 (11.83 to 18.79)	<0.001	6.08 (2.89 to 12.81)	<0.001	5.18 (2.21 to 12.16)	<0.001
Trend test		<0.001		<0.001		<0.001		<0.001		<0.001
Baseline symptom severity	−0.30 (−0.67 to 0.06)	0.101	−0.40 (−0.88 to 0.08)	0.104	0.47 (−0.02 to 0.96)	0.062	1.03 (0.95 to 1.13)	0.469	1.06 (0.98 to 1.16)	0.164
Follow-up symptom severity	−0.68 (−0.79 to −0.56)	<0.001	−0.70 (−0.86 to −0.53)	<0.001	1.19 (1.06 to 1.32)	<0.001	1.09 (1.05 to 1.13)	<0.001	1.10 (1.06 to 1.14)	<0.001
	Model I		Model I		Model I		Model I		Model I	
Trajectory groups										
Persistent-low	0 (Ref)		0 (Ref)		0 (Ref)		1 (Ref)		1 (Ref)	
High-stable	−4.83 (−7.31 to −2.35)	<0.001	−9.49 (−12.84 to −6.14)	<0.001	11.93 (8.68 to 15.17)	<0.001	3.83 (1.75 to 8.39)	0.001	6.74 (2.89 to 15.71)	<0.001
Low-rapid-progression	−10.92 (−13.58 to −8.27)	<0.001	−11.67 (−15.26 to −8.09)	<0.001	15.79 (12.32 to 19.27)	<0.001	5.52 (2.55 to 11.94)	< 0.001	5.02 (2.09 to 12.09)	<0.001
Trend test		<0.001		<0.001		<0.001		< 0.001		<0.001
Baseline symptom severity	−0.26 (−0.62 to 0.10)	0.160	−0.41 (−0.89 to 0.06)	0.090	0.47 (−0.02 to 0.97)	0.060	1.04 (0.95 to 1.13)	0.409	1.07 (0.98 to 1.17)	0.108
Follow-up symptom severity	−0.66 (−0.78 to −0.55)	<0.001	−0.71 (−0.88 to −0.54)	<0.001	1.18 (1.05 to 1.32)	<0.001	1.09 (1.05 to 1.14)	<0.001	1.11 (1.06 to 1.16)	<0.001
	Model II		Model II		Model II		Model II		Model II	
Trajectory groups										
Persistent-low	0 (Ref)		0 (Ref)		0 (Ref)		1 (Ref)		1 (Ref)	
High-stable	−4.36 (−6.87 to −1.85)	0.001	−9.52 (−12.93 to −6.12)	<0.001	11.10 (7.84 to 14.37)	<0.001	4.49 (1.98 to 10.19)	<0.001	7.34 (3.04 to 17.77)	<0.001
Low-rapid-progression	−10.80 (−13.45 to −8.14)	<0.001	−11.59 (−15.19 to −7.98)	<0.001	15.65 (12.19 to 19.10)	<0.001	5.92 (2.70 to 12.98)	<0.001	5.01 (2.06 to 12.19)	<0.001
Trend test		<0.001		<0.001		<0.001		<0.001		<0.001
Baseline symptom severity	−0.23 (−0.59 to 0.13)	0.216	−0.42 (−0.90 to 0.07)	0.093	0.39 (−0.10 to 0.89)	0.120	1.05 (0.95 to 1.14)	0.340	1.06 (0.97 to 1.16)	0.172
Follow-up symptom severity	−0.65 (−0.77 to −0.53)	<0.001	−0.72 (−0.88 to −0.55)	<0.001	1.17 (1.04 to 1.31)	<0.001	1.11 (1.07 to 1.16)	<0.001	1.12 (1.07 to 1.17)	<0.001
	Model III		Model III		Model III		Model III		Model III	
Trajectory groups										
Persistent-low	0 (Ref)		0 (Ref)		0 (Ref)		1 (Ref)		1 (Ref)	
High-stable	−2.41 (−5.18 to 0.36)	0.089	−5.20 (−8.75 to −1.66)	0.004	8.20 (4.59 to 11.80)	<0.001	1.93 (0.65 to 5.68)	0.235	4.35 (1.44 to 13.18)	0.009
Low-rapid-progression	−9.99 (−12.65 to −7.34)	<0.001	−9.53 (−12.92 to −6.13)	<0.001	14.44 (10.98 to 17.89)	<0.001	4.75 (1.96 to 11.48)	0.001	4.21 (1.57 to 11.27)	0.004
Trend test		<0.001		<0.001		<0.001		0.001		0.003
Baseline symptom severity	0.17 (−0.23 to 0.57)	0.410	0.33 (−0.17 to 0.83)	0.200	−0.30 (−0.84 to 0.24)	0.270	0.88 (0.76 to 1.03)	0.103	0.93 (0.82 to 1.06)	0.256
Follow-up symptom severity	−0.60 (−0.72 to −0.48)	<0.001	−0.57 (−0.73 to −0.41)	<0.001	1.10 (0.96 to 1.24)	<0.001	1.09 (1.05 to 1.14)	<0.001	1.10 (1.05 to 1.15)	<0.001

OR, odds ratio; 95% CI, 95% confidence interval; PCS, Physical Component Summary; MCS, Mental Component Summary; HADS, Hospital Anxiety and Depression Scale; HADS-A, Hospital Anxiety and Depression Scale–anxiety subscale; HADS-D, Hospital Anxiety and Depression Scale–depression subscale; DASH, Disabilities of the Arm, Shoulder, and Hand questionnaire; BMI, body mass index; ALND, axillary lymph node dissection.

Model I was adjusted for age, educational level, marital status, employment status, BMI, and follow-up time point (12 vs 24 months).

Model II was adjusted for all covariates in Model I and additionally for ALND affected dominant arm, chemotherapy, taxane-based chemotherapy, and radiotherapy.

Model III was adjusted for all covariates in Model II and additionally for baseline SF-36v2 PCS, MCS, HADS, and DASH scores, and weight change from baseline measured at the same follow-up time point as the outcome.

Outcomes represent each participant's last available follow-up PRO assessment (12-month PRO for participants who completed only the 12-month visit; 24-month PRO for participants who completed the 24-month visit). Trajectory groups served as the primary exposure; baseline and follow-up BCLE-SEI symptom severity were analyzed in separate complementary models. Conventional standard errors were used in the primary models; a sensitivity analysis comparing conventional and Huber–White robust standard errors is presented in Supplementary Table S3. Repeated measures were handled separately in the mixed-effects models (Supplementary Table S8).

### Subgroup analysis

Stratified analyses examined potential effect modification of the association between the lymphedema symptom trajectories and PROs (Supplementary Table S9). No significant interactions were observed in any subgroups after stratifying by age, educational level, marital status, employment status, BMI, ALND affected dominant arm, taxane-based chemotherapy, or radiotherapy, except for a radiotherapy × HADS-D interaction (*P* = 0.036) (Fig. 4). These subgroup analyses are exploratory and should be interpreted with caution given the small number of participants in some strata (e.g., low-rapid-progression without radiotherapy, *n* = 7) and the number of interaction tests performed.

**Fig. 4:**
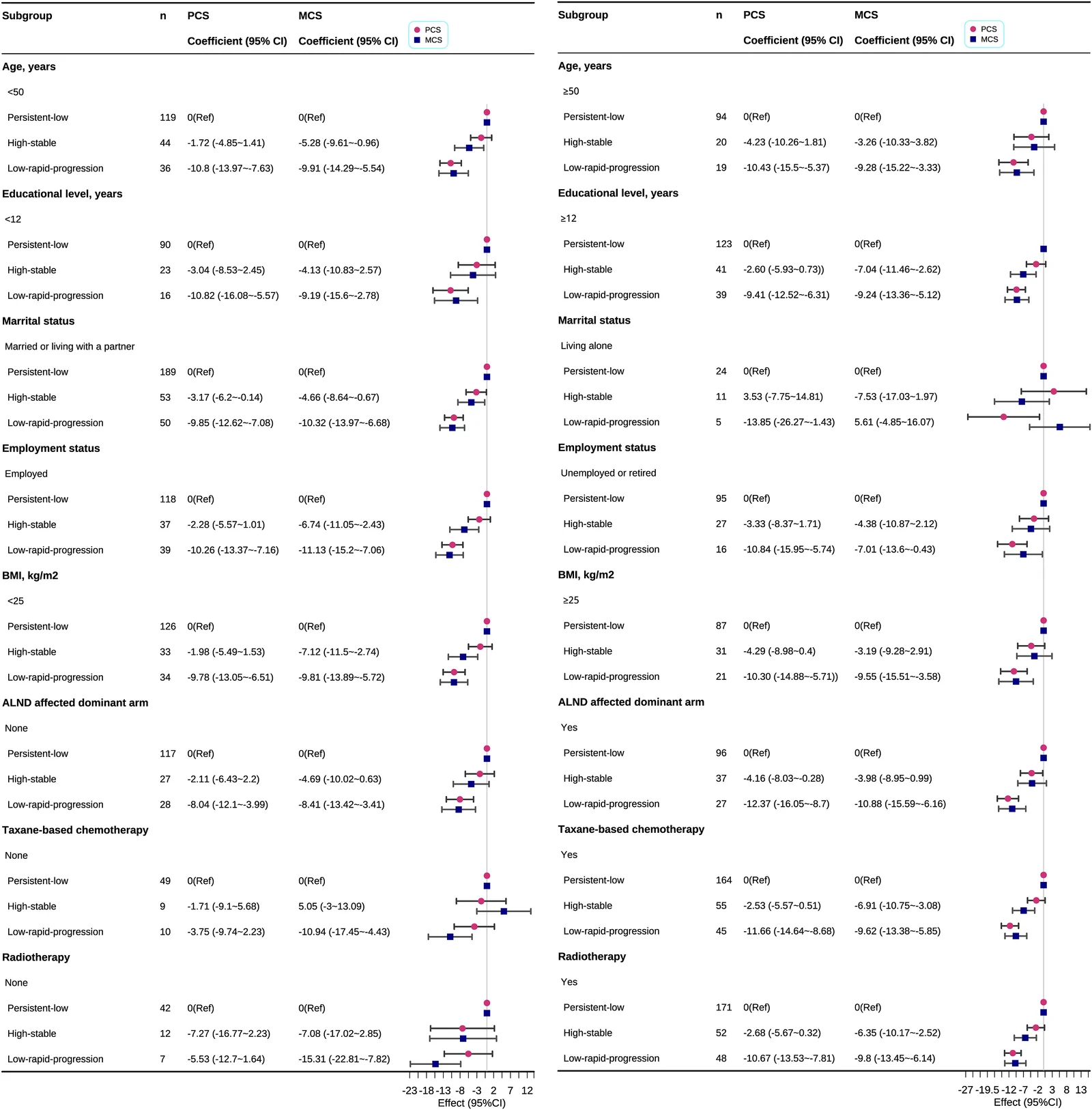

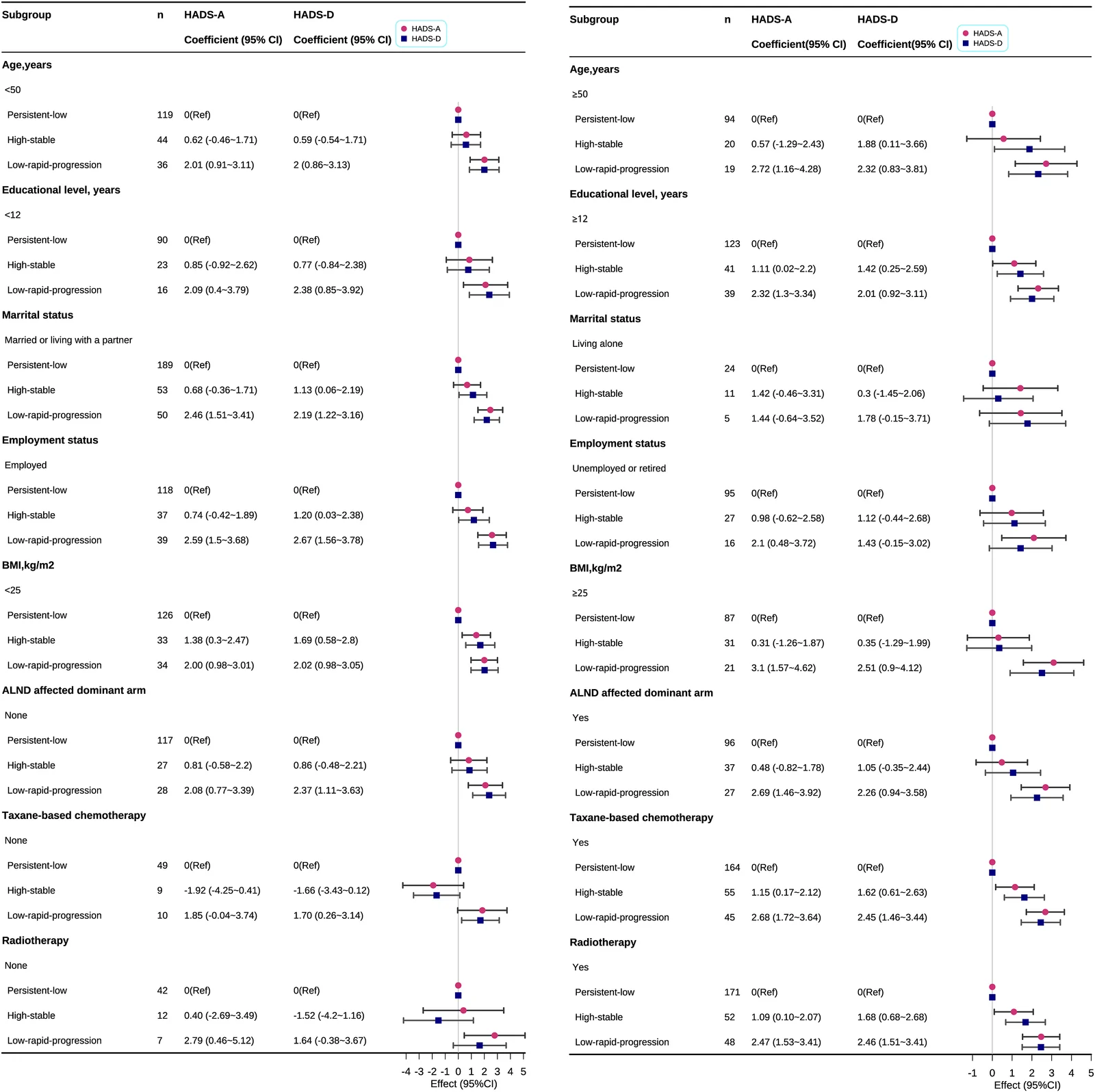

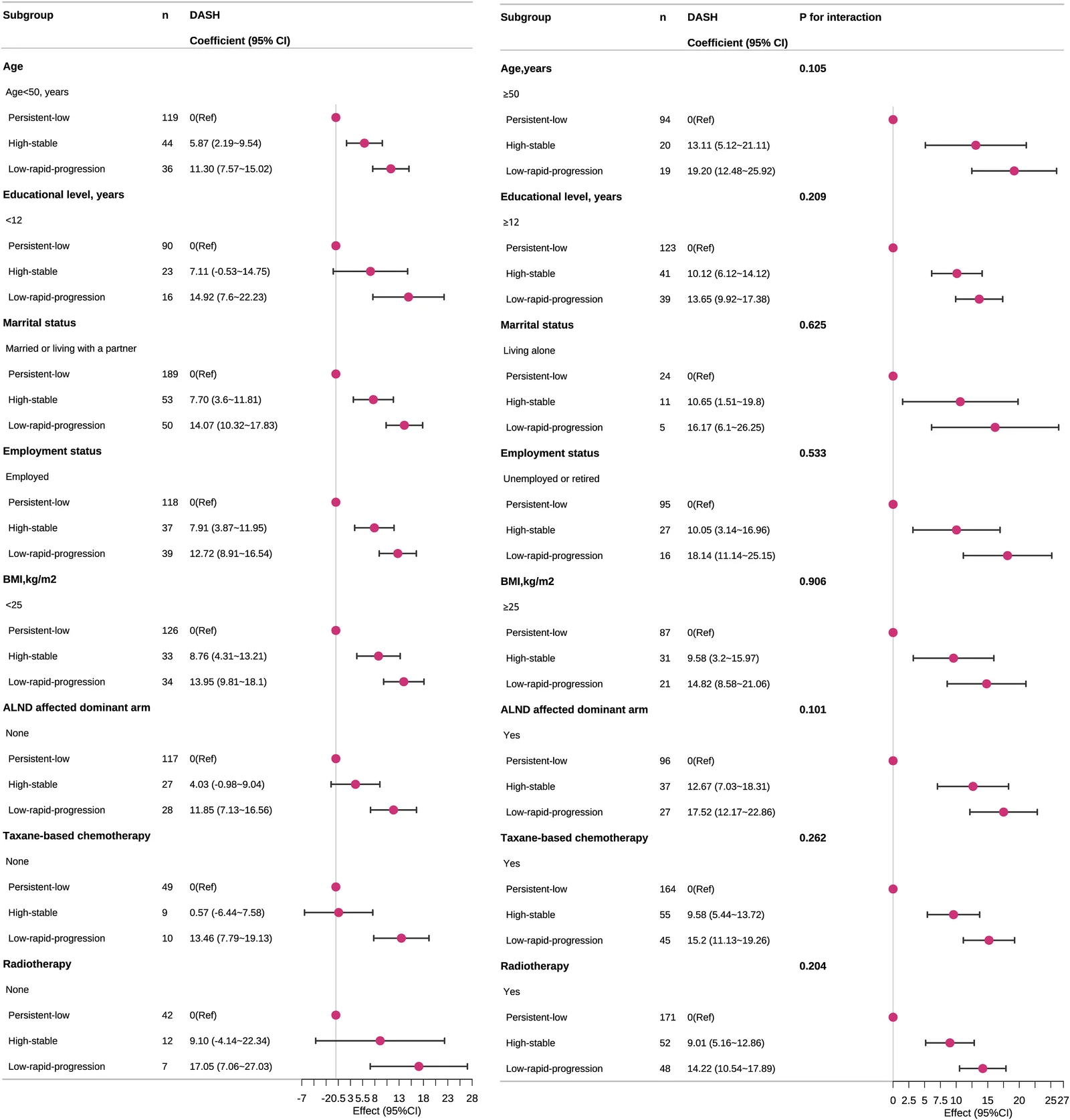
Associations between lymphedema symptom trajectories and PROs by subgroup. In each stratified analysis, models were adjusted for all covariates other than the stratification variable (age, educational level, marital status, employment status, BMI, follow-up time point, ALND affected dominant arm, chemotherapy, taxane-based chemotherapy, radiotherapy, baseline PCS, MCS, HADS, DASH, and weight change from baseline). HADS-A and HADS-D were analyzed as continuous scores (0–21) in the subgroup analyses; all effect estimates are reported as regression coefficients (*β*) with 95% confidence intervals (CIs). 95% CI, 95% confidence interval; BMI, body mass index; PROs, patient-reported outcomes; PCS, Physical Component Summary; MCS, Mental Component Summary; HADS-A, Hospital Anxiety and Depression Scale–anxiety subscale; HADS-D, Hospital Anxiety and Depression Scale–depression subscale; DASH, Disabilities of the Arm, Shoulder, and Hand questionnaire.

## Discussion

### Main findings

This prospective cohort study identified three lymphedema symptom trajectories: persistent-low, high-stable, and low-rapid-progression. PROs followed distinct longitudinal patterns across these groups. Compared with the persistent-low group, the low-rapid-progression group had poorer QoL, greater risks of anxiety and depression, and more severe upper limb impairment. The study provided no prophylactic lymphedema interventions, such as compression therapy, manual lymphatic drainage, or structured rehabilitation; all participants received uniform surveillance, with referral to a certified lymphedema therapist upon detection of clinically significant swelling, as part of standard postoperative care. Treatment received after referral was not recorded and may have influenced symptoms and PROs observed at subsequent assessments. Thus, the observed trajectory–PRO associations describe the course of symptom burden after ALND under uniform surveillance and indication-based referral; however, the potential influence of post-referral treatment cannot be excluded.

#### Lymphedema symptom burden

Subjective arm swelling was more prevalent than objectively measured lymphedema during follow-up, affecting 44.0% and 9.3% of participants, respectively. This nearly fivefold difference suggests that patient-reported symptoms may capture early or subclinical changes, consistent with ISL Stage 0, before volumetric changes become detectable.^11^ Even with a standardized protocol, arm circumference measurement may be insufficiently sensitive to early fluid shifts and soft-tissue changes. Similarly, in a prospective cohort of 281 patients treated with ALND, Heller et al. reported perceived arm swelling in 55% of participants but measured lymphedema in 20% at 2 years.^32^ Symptom assessment captures the patient's experience and may provide early warning, whereas objective measurement provides the specificity and precision needed to monitor progression. Both the 2023 ISL Consensus Document and the American Physical Therapy Association (APTA) Clinical Practice Guideline for BCRL support integrating subjective and objective assessment.^11^^,^^33^ The APTA guideline further indicates that prospective surveillance for subclinical lymphedema in high-risk populations may improve outcomes (Grade C).^33^

Symptom burden varied markedly at 24 months, with 16.6% of participants following a low-rapid-progression trajectory. A previous randomized trial also identified three symptom trajectories over 24 months, namely stable, slight increase or decrease, and increasing, with 9% of participants in the increasing group.^22^ Smoot et al. used latent class growth analysis to examine bioimpedance resistance ratios in 380 women over 12 months and identified three limb-volume classes comprising 37.9%, 46.8%, and 15.3% of participants.^21^ Ridner et al. jointly evaluated L-Dex, arm volume, and symptom-cluster trajectories in 508 participants over 24 months.^22^ By contrast, the present study used GBTM to assess BCLE-SEI symptom trajectories in 332 participants after unilateral ALND over 24 months and identified persistent-low (64.2%), high-stable (19.3%), and low-rapid-progression (16.6%) groups. Differences in surgical populations, constructs, sample sizes, and follow-up schedules likely account for the variation. Symptoms in the progressive group increased after surgery, declined somewhat before the 24-month assessment, and remained above baseline at 24 months, supporting prospective surveillance for at least 24 months after surgery.

#### Associations between lymphedema symptom burden and patient-reported outcomes

Consistent with previous evidence, PROs were impaired after breast cancer surgery, and greater lymphedema symptom burden was associated with more severe functional impairment.^34^^,^^35^ Compared with the persistent-low group, the low-rapid-progression group had sustained deficits in physical health-related QoL and upper limb function through 24 months, without return to preoperative levels. This is consistent with a prospective study in which physical QoL declined from 6 months after ALND and remained below the preoperative level at 24 months.^32^ Upper limb morbidity is also common among breast cancer survivors; one study reported shoulder pain and stiffness in up to 43% and 36% of patients, respectively.^36^

After adjustment for baseline outcome scores and demographic and clinical covariates, lymphedema symptom trajectories remained independently associated with PROs. Previous studies reported poorer QoL, greater anxiety and depression, and worse upper limb or shoulder function among breast cancer survivors with moderate-to-severe lymphedema symptoms than among those with minimal or no symptoms.^34^^,^^37^, ^38^, ^39^ However, longitudinal evidence comparing PROs across distinct symptom trajectories remains limited. The present study extends this literature by characterizing associations between lymphedema symptom trajectories and multiple PRO domains after accounting for measured demographic and clinical factors.

Although both non-persistent-low trajectories were independently associated with adverse PROs, their clinical patterns differed. The high-stable group entered the study with greater symptom burden and poorer PROs, consistent with preoperative QoL deficits reported in ALND cohorts.^32^^,^^34^ Symptoms remained elevated but relatively stable through 24 months, whereas PCS scores improved during follow-up. This pattern may reflect pre-existing comorbidity or functional limitation, with adaptation and routine supportive care. By contrast, the low-rapid-progression group began with low BCLE-SEI scores but experienced substantial postoperative deterioration in PCS and DASH scores that persisted through 24 months, consistent with prolonged physical impairment after axillary surgery.^32^ For the psychological domains (MCS, HADS-A, and HADS-D), the group-by-time interactions were no longer statistically significant by 24 months, although MCS remained lower and the odds of HADS-A and HADS-D ≥ 8 remained higher in the low-rapid-progression group than in the persistent-low group. This contrasts with the persistent impairment of the physical domains (PCS and DASH) and parallels the physical–psychological dissociation reported in previous ALND cohorts.^32^ These findings suggest that symptom progression, rather than baseline severity alone, is closely associated with sustained physical impairment; however, the observational design does not permit causal inference.

Because baseline PROs differed across trajectory groups, the direction of the observed associations cannot be established; poorer baseline PROs may have predisposed some participants to worsening symptom trajectories. Nevertheless, a sensitivity analysis restricted to 12 swelling- or edema-specific symptoms produced associations consistent with the primary analysis, supporting the robustness of the findings to potential measurement overlap (Supplementary Table S5). Moreover, E-value analyses indicated that an unmeasured confounder would need to be associated with both the trajectory group and the outcome by a risk ratio of at least approximately 2.4–3.6, based on the conservative confidence-interval limits closest to the null (point-estimate E-values ranged from approximately 3.3 to 9.0), to fully explain away the observed associations (Supplementary Table S6).

### Implications for nursing practice and research

These findings have several implications for oncology nursing. Lymphedema symptoms should be assessed systematically with a validated instrument, such as the BCLE-SEI, preoperatively and at 12 and 24 months after ALND. The discrepancy between subjective and objective lymphedema, 44.0% versus 9.3%, indicates that symptoms warrant clinical evaluation even when arm volume remains below the 10% RVC threshold. A meaningful increase from baseline should prompt evaluation by a certified lymphedema therapist, and patients should receive education on monitoring and reporting early symptoms such as heaviness, tightness, and swelling. The divergent trajectories also support risk-stratified surveillance, particularly for patients with initially low symptoms followed by rapid postoperative progression and sustained impairment, who may require closer monitoring. It should be noted that trajectory-group membership was derived from the complete longitudinal symptom data after follow-up was completed and therefore cannot be predicted at baseline; serial symptom monitoring identifies patients with worsening symptoms rather than predicting their eventual trajectory group. Prophylactic measures, including compression sleeves, may be considered when clinically appropriate.^40^^,^^41^

### Limitations

This study has several limitations. First, symptoms and PROs were assessed only at baseline and at 12 and 24 months, consistent with previous trajectory studies.^21^^,^^22^ These annual assessments limited temporal resolution; assessments at 3- to 6-month intervals would better define the timing and rate of progression, particularly in the low-rapid-progression group. Second, 51 participants who died, experienced disease progression, or were lost to follow-up were excluded. Compared with the analytic cohort, these participants were older, had lower educational level, were less often employed, had more comorbidities and more positive lymph nodes, although pathological tumor-node-metastasis stage and chemotherapy rates were similar (Supplementary Table S10). Their exclusion may have influenced the trajectory–PRO associations. These sensitivity analyses support the consistency of the findings but do not establish that measurement overlap or attrition had no influence on the estimates. In addition, only 118 of 332 participants (35.5%) completed the 24-month assessment, primarily because of staggered enrollment. The 67 participants who did not return after 12 months were comparable with completers for all 12-month symptom and PRO measures (Supplementary Table S1, Panel D), and a complete-case sensitivity analysis confirmed the direction of the primary associations (Supplementary Table S4). The limited 24-month data reduced the precision of later estimates. Third, the sample size was determined for the parent study rather than the trajectory analysis. The low-rapid-progression group included only 55 participants, limiting power for subgroup analyses; between-group differences should be interpreted with caution. Fourth, the interaction between radiotherapy and trajectory group for HADS-D was identified in these exploratory stratified analyses and may reflect the small no-radiotherapy subgroup, which included only 7 low-rapid-progression participants, rather than true effect modification. This finding requires confirmation in larger cohorts. Finally, treatment received after referral to a certified lymphedema therapist, physical activity level, and other lifestyle factors were not collected; these unmeasured factors may have influenced the observed symptoms and PROs, and residual confounding cannot be excluded despite adjustment for measured demographic and clinical covariates. E-value analyses (Supplementary Table S6) indicated that, based on the confidence-interval limits closest to the null, an unmeasured confounder would need a risk ratio of at least approximately 2.4–3.6 with both the trajectory group and the outcome to fully explain away the observed associations.

## Conclusions

Lymphedema symptom burden was associated with poorer PROs among breast cancer survivors after ALND. Compared with the persistent-low group, the low-rapid-progression group had sustained deficits in physical QoL and ipsilateral upper limb function throughout follow-up. These findings support longitudinal symptom surveillance and justify interventional studies to determine whether earlier symptom control improves PROs.

## CRediT authorship contribution statement

**Cuiju Wen**: Writing – original draft, Conceptualization, Data curation, Formal analysis, Methodology. **Hongjuan Sun**: Writing – review & editing, Conceptualization, Data curation, Supervision. **Xiang Li**: Writing – review & editing, Conceptualization, Data curation. **Jingyan Xu**: Writing – review & editing, Conceptualization. **Jie Zhang**: Writing – review & editing, Data curation. **Lize Wang**: Writing – review & editing, Conceptualization. **Yang Yang**: Writing – review & editing, Conceptualization. **Zhaoqing Fan**: Writing – review & editing, Writing – original draft, Conceptualization, Formal analysis, Supervision. **Xinying Yu**: Writing – review & editing, Writing – original draft, Funding acquisition, Supervision, Formal analysis, Conceptualization. All authors have read and approved the final manuscript.

## Ethics statement

The study was approved by the Research Ethics Committee of Peking University Cancer Hospital (Approval No. 2022KT78) and was conducted in accordance with the Declaration of Helsinki and its subsequent amendment. All participants provided written informed consent.

## Data availability

The data that support the findings of this study are available from the corresponding author, upon reasonable request.

## Declaration of generative AI and AI-assisted technologies in the writing process

During the preparation of this work, the authors used DeepSeek V4 (OpenCode Go) for language editing and to improve the readability of the manuscript. After using this tool, the authors reviewed and edited the content as needed and take full responsibility for the content of the publication.

## Funding

This work was supported by the Science Foundation of Peking University Cancer Hospital (Grant No. 2022–12). The funders had no role in the study design; data collection, analysis, or interpretation; preparation of the manuscript; or the decision to submit the article for publication.

## Declaration of competing interest

The authors declare that they have no competing interests relevant to this work.
