# Supplementary material for: Lymphedema symptom trajectories and patient-reported outcomes after axillary lymph node dissection for breast cancer: A prospective cohort study
Source: Asia Pac J Oncol Nurs. 2026 Oct 5;13:101041. doi: 10.1016/j.apjon.2026.101041 (PMC13740882; doi:10.1016/j.apjon.2026.101041)
Supplement: Supplementary file 1 [file mmc2.pdf]

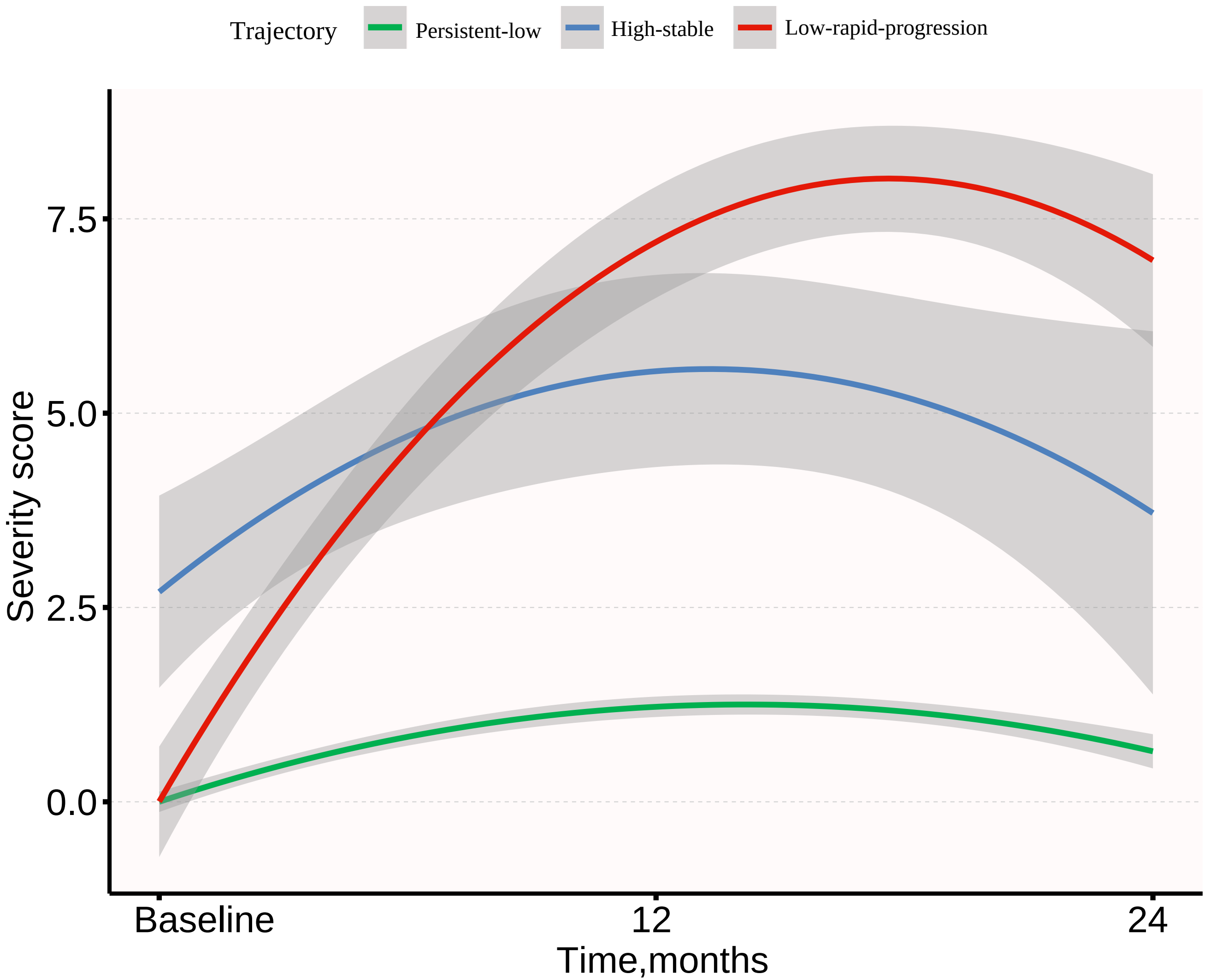

Trajectory
Persistent-low
High-stable
Low-rapid-progression
Severity score
7.5
5.0
2.5
0.0
Baseline
12
24
Time,months
