# Supplementary material for: Lymphedema symptom trajectories and patient-reported outcomes after axillary lymph node dissection for breast cancer: A prospective cohort study
Source: Asia Pac J Oncol Nurs. 2026 Oct 5;13:101041. doi: 10.1016/j.apjon.2026.101041 (PMC13740882; doi:10.1016/j.apjon.2026.101041)
Supplement: Supplementary file 2 [file mmc1.docx]

**Supplementary Tables:**

Table S1. Data availability across the three longitudinal assessment time points (n = 332)

Table S2. Univariable associations between covariates and PROs

Table S3. Sensitivity analysis comparing conventional versus Huber–White robust standard errors for the association between lymphedema symptom trajectories and PROs (n = 332)

Table S4. Sensitivity analysis: association between lymphedema symptom trajectories and PROs restricted to participants with complete data at all three time points (n = 117), compared with the primary analysis (n = 332)

Table S5. Sensitivity analysis: association of lymphedema-specific symptom trajectories (12-item subset) with PROs

Table S6. E-value analysis for unmeasured confounding (low-rapid-progression vs. persistent-low trajectory, Model III)

Table S7. Prevalence and severity of lymphedema-related symptoms (n = 332)

Table S8. Mixed-effects model for repeated measures of PROs

Table S9. Association between the lymphedema symptom trajectories and PROs across subgroups

Table S10. Baseline characteristics of participants in the analytic cohort (n = 332) versus those excluded (n = 51)

**Table S1. Data availability across the three longitudinal assessment time points (n = 332)**

Panel A. Assessment completion by time point

| Time point | Available, n | Missing, n | Completion, % |
| --- | --- | --- | --- |
| Baseline (preoperative) | 332 | 0 | 100.0 |
| 12 months post-ALND | 331 | 1 | 99.7 |
| 24 months post-ALND | 118 | 214 | 35.5 |

Panel B. Longitudinal data patterns and reasons for missing 24-month assessments

| Pattern | n | % |
| --- | --- | --- |
| All three time points completed | 117 | 35.2 |
| Baseline + 12 months only-not yet due for 24-month visit | 147 | 44.3 |
| Baseline + 12 months only-did not return (transferred care) | 67 | 20.2 |
| Baseline + 24 months only (missed 12-month visit) | 1 | 0.3 |

Panel C. Item-level missingness by instrument (within completed assessments)

| Instrument | Baseline (n = 332) | 12 months (n = 331) | 24 months (n = 118) |
| --- | --- | --- | --- |
| BCLE-SEI, % | <1.0 | <1.0 | <1.0 |
| SF-36v2, % | <1.0 | <1.0 | <1.0 |
| HADS, % | <1.0 | <1.0 | <1.0 |
| DASH, % | <1.0 | <1.0 | <1.0 |

Panel D. Comparison of 12-month symptom scores and PROs between participants who completed versus did not return for the 24-month visit (n = 184)

| Variables | Total (n = 184) | Completers (n = 117) | Non-returners (n = 67) | *p*-value |
| --- | --- | --- | --- | --- |
| BCLE-SEI symptom score, Median (IQR) | 4.0 (1.0, 9.2) | 4.0 (1.0, 9.0) | 5.0 (1.0, 9.5) | 0.362 |
| PCS, Mean (SD) | 45.6 (10.0) | 45.9 (9.9) | 45.0 (10.2) | 0.525 |
| MCS, Mean (SD) | 48.6 (13.6) | 49.0 (13.3) | 47.8 (14.1) | 0.556 |
| HADS-A, Median (IQR) | 3.5 (1.0, 6.0) | 4.0 (1.0, 7.0) | 3.0 (2.0, 6.0) | 0.704 |
| HADS-D, Median (IQR) | 2.0 (1.0, 5.0) | 2.0 (1.0, 6.0) | 2.0 (1.0, 3.5) | 0.392 |
| DASH, Median (IQR) | 9.6 (2.5, 19.2) | 9.2 (2.5, 18.3) | 10.0 (2.1, 19.6) | 0.771 |
| HADS-A ≥ 8, n (%) | 31 (16.8) | 21 (17.9) | 10 (14.9) | 0.598 |
| HADS-D ≥ 8, n (%) | 28 (15.2) | 20 (17.1) | 8 (11.9) | 0.349 |

Notes: In Panel B, participants classified as "not yet due for 24-month visit" underwent surgery between August 2023 and July 2024 and had therefore not reached the 24-month milestone at the time of data analysis. Participants in the "did not return" category had completed ≥24 months since surgery but were lost to the 24-month assessment because their postoperative follow-up had been transferred to other hospitals. The single participant in "Baseline + 24 months only" attended the 24-month visit but missed the 12-month assessment. In Panel C, item-level missingness within completed assessments was uniformly below 1% across all instruments and time points.

Abbreviations: BCLE-SEI, Breast Cancer and Lymphedema Symptom Experience Index; SF-36v2, 36-Item Short Form Health Survey version 2; HADS, Hospital Anxiety and Depression Scale; DASH, Disabilities of the Arm, Shoulder, and Hand questionnaire; ALND, axillary lymph node dissection; PCS, Physical Component Summary; MCS, Mental Component Summary; HADS-A, Hospital Anxiety and Depression Scale–anxiety subscale; HADS-D, Hospital Anxiety and Depression Scale–depression subscale; IQR, interquartile range; SD, standard deviation.

**Table S2. Univariable associations between covariates and PROs**

| Item | PCS  *β* (95% CI) | *p*-value | MCS  *β* (95% CI) | *p*-value | DASH  *β* (95% CI) | *p*-value | HADS-A ≥ 8  OR (95% CI) | *p*-value | HADS-D ≥ 8  OR (95% CI) | *p*-value |
| --- | --- | --- | --- | --- | --- | --- | --- | --- | --- | --- |
| Age (years) | -0.07  (-0.18 to 0.05) | 0.253 | 0.07  (-0.07 to 0.22) | 0.324 | 0.02  (-0.14 to 0.17) | 0.847 | 0.97 (0.94 to 1.01) | 0.100 | 0.99 (0.95 to 1.02) | 0.503 |
| Educational level (years), n (%) |  |  |  |  |  |  |  |  |  |  |
| <12 | Reference |  | Reference |  | Reference |  | Reference |  | Reference |  |
| ≥12 | -2.32  (-4.48 to -0.16) | 0.035 | -0.40  (-3.28 to 2.49) | 0.787 | 3.50 (0.56 to 6.44) | 0.020 | 2.06 (1.05 to 4.03) | 0.036 | 1.12 (0.57 to 2.20) | 0.750 |
| Marital status, n (%) |  |  |  |  |  |  |  |  |  |  |
| Married or living with a partner | Reference |  | Reference |  | Reference |  | Reference |  | Reference |  |
| Living alone | -3.25  (-6.49 to -0.02) | 0.049 | 5.95 (1.68 to 10.22) | 0.006 | 0.32  (-4.12 to 4.76) | 0.887 | 0.26 (0.06 to 1.12) | 0.070 | 0.34 (0.08 to 1.47) | 0.149 |
| Employment status, n (%) |  |  |  |  |  |  |  |  |  |  |
| Employed | Reference |  | Reference |  | Reference |  | Reference |  | Reference |  |
| Unemployed | -0.08  (-3.23 to 3.06) | 0.958 | 1.04  (-3.13 to 5.21) | 0.625 | -3.02  (-7.30 to 1.26) | 0.166 | 0.62 (0.25 to 1.57) | 0.315 | 0.60 (0.20 to 1.81) | 0.366 |
| Retired | -0.56  (-3.02 to 1.89) | 0.652 | 0.45  (-2.81 to 3.71) | 0.784 | 0.09  (-3.25 to 3.43) | 0.956 | 0.41 (0.18 to 0.92) | 0.030 | 0.89 (0.42 to 1.89) | 0.758 |
| BMI (kg/m^2^) | -0.07  (-0.36 to 0.22) | 0.645 | 0.10 (-0.29 to 0.48) | 0.623 | -0.30  (-0.69 to 0.10) | 0.142 | 1.00  (0.92 to 1.09) | 0.991 | 0.99 (0.90 to 1.08) | 0.813 |
| ALND affected dominant arm, n (%) |  |  |  |  |  |  |  |  |  |  |
| None | Reference |  | Reference |  | Reference |  | Reference |  | Reference |  |
| Yes | -0.59  (-2.72 to 1.53) | 0.582 | -1.45  (-4.26 to 1.36) | 0.310 | 1.60  (-1.29 to 4.49) | 0.276 | 0.79 (0.43 to 1.43) | 0.433 | 1.28 (0.67 to 2.47) | 0.455 |
| Affected side, n (%) |  |  |  |  |  |  |  |  |  |  |
| Left | Reference |  | Reference |  | Reference |  | Reference |  | Reference |  |
| Right | -0.39  (-2.51 to 1.73) | 0.718 | -1.86  (-4.67 to 0.95) | 0.193 | 0.42  (-2.48 to 3.31) | 0.776 | 0.68 (0.37 to 1.25) | 0.211 | 1.34 (0.69 to 2.58) | 0.384 |
| cTNM stage, n (%) |  |  |  |  |  |  |  |  |  |  |
| I | Reference |  | Reference |  | Reference |  | Reference |  | Reference |  |
| II | 1.22  (-10.01 to 12.45) | 0.831 | 6.95  (-7.92 to 21.82) | 0.359 | -7.98  (-23.26 to 7.30) | 0.305 | 0.37 (0.03 to 4.14) | 0.418 | — | 0.987 |
| III | 0.65  (-10.84 to 12.14) | 0.912 | 7.65  (-7.56 to 22.86) | 0.323 | -6.44  (-22.06 to 9.19) | 0.419 | 0.31 (0.02 to 3.90) | 0.366 | — | 0.987 |
| pTNM stage, n (%) |  |  |  |  |  |  |  |  |  |  |
| 0 | Reference |  | Reference |  | Reference |  | Reference |  | Reference |  |
| I | -2.27  (-6.34 to 1.79) | 0.272 | -3.52  (-8.91 to 1.87) | 0.200 | -0.31  (-5.86 to 5.25) | 0.913 | 1.60 (0.48 to 5.32) | 0.445 | 2.31 (0.71 to 7.51) | 0.165 |
| II | -1.65  (-4.39 to 1.08) | 0.236 | -1.48  (-5.11 to 2.15) | 0.423 | 0.95  (-2.78 to 4.69) | 0.616 | 1.84 (0.79 to 4.31) | 0.159 | 1.45 (0.58 to 3.65) | 0.428 |
| III | -1.79  (-4.87 to 1.28) | 0.252 | -1.38  (-5.46 to 2.70) | 0.506 | 0.47  (-3.74 to 4.67) | 0.827 | 1.65 (0.64 to 4.23) | 0.298 | 1.41 (0.51 to 3.91) | 0.511 |
| Breast surgery, n (%) |  |  |  |  |  |  |  |  |  |  |
| BCS | Reference |  | Reference |  | Reference |  | Reference |  | Reference |  |
| Mastectomy | -2.12  (-4.45 to 0.22) | 0.076 | -1.20  (-4.31 to 1.91) | 0.448 | 2.96  (-0.22 to 6.14) | 0.068 | 2.06 (0.96 to 4.42) | 0.064 | 1.28 (0.60 to 2.72) | 0.523 |
| No. of nodes removed | 0.00  (-0.13 to 0.13) | 0.979 | 0.06  (-0.11 to 0.23) | 0.476 | -0.01  (-0.18 to 0.17) | 0.941 | 1.02 (0.99 to 1.05) | 0.277 | 0.99 (0.94 to 1.03) | 0.508 |
| No. of positive nodes | -0.09  (-0.24 to 0.07) | 0.266 | -0.01  (-0.21 to 0.20) | 0.935 | 0.07  (-0.14 to 0.28) | 0.500 | 1.01 (0.97 to 1.05) | 0.666 | 0.97 (0.90 to 1.04) | 0.432 |
| Chemotherapy, n (%) |  |  |  |  |  |  |  |  |  |  |
| None | Reference |  | Reference |  | Reference |  | Reference |  | Reference |  |
| Neoadjuvant chemotherapy | -1.29  (-4.95 to 2.37) | 0.488 | 1.10  (-3.75 to 5.95) | 0.656 | 2.21  (-2.77 to 7.19) | 0.383 | 0.79 (0.30 to 2.07) | 0.634 | 1.53 (0.44 to 5.32) | 0.507 |
| Adjuvant chemotherapy | -1.75  (-5.79 to 2.30) | 0.396 | 1.04  (-4.32 to 6.41) | 0.702 | 2.97  (-2.53 to 8.48) | 0.289 | 0.69 (0.23 to 2.06) | 0.507 | 1.07 (0.27 to 4.33) | 0.920 |
| Taxane-based chemotherapy, n (%) |  |  |  |  |  |  |  |  |  |  |
| None | Reference |  | Reference |  | Reference |  | Reference |  | Reference |  |
| Neoadjuvant | -1.38  (-4.11 to 1.34) | 0.319 | -1.44  (-5.08 to 2.20) | 0.437 | 3.32 (-0.37 to 7.01) | 0.078 | 0.94 (0.44 to 2.00) | 0.865 | 1.08 (0.46 to 2.54) | 0.863 |
| Adjuvant | -3.52  (-6.68 to -0.36) | 0.029 | -1.58  (-5.80 to 2.63) | 0.461 | 6.89 (2.62 to 11.16) | 0.002 | 0.90 (0.37 to 2.19) | 0.819 | 1.06 (0.39 to 2.85) | 0.914 |
| Radiotherapy, n (%) |  |  |  |  |  |  |  |  |  |  |
| None | Reference |  | Reference |  | Reference |  | Reference |  | Reference |  |
| Yes | -1.68  (-4.41 to 1.05) | 0.228 | -0.77  (-4.40 to 2.86) | 0.677 | 0.44  (-3.30 to 4.17) | 0.818 | 1.25 (0.55 to 2.81) | 0.591 | 1.72 (0.64 to 4.57) | 0.280 |
| Endocrine therapy, n (%) |  |  |  |  |  |  |  |  |  |  |
| None | Reference |  | Reference |  | Reference |  | Reference |  | Reference |  |
| TAM | 2.49  (-1.25 to 6.24) | 0.191 | 3.22  (-1.74 to 8.17) | 0.202 | -4.06  (-9.16 to 1.04) | 0.119 | 0.45 (0.12 to 1.68) | 0.236 | 0.49 (0.10 to 2.37) | 0.374 |
| AI | 0.03  (-2.43 to 2.49) | 0.982 | -1.01  (-4.26 to 2.24) | 0.543 | -1.53  (-4.88 to 1.82) | 0.369 | 1.04 (0.53 to 2.06) | 0.899 | 1.50 (0.68 to 3.30) | 0.318 |
| CCI | -0.55 (-1.47 to 0.37) | 0.238 | 0.35  (-0.86 to 1.57) | 0.567 | 0.79 (-0.46 to 2.04) | 0.212 | 0.88 (0.67 to 1.15) | 0.353 | 1.01 (0.76 to 1.35) | 0.921 |
| Lymphedema (RVC ≥ 10%) |  |  |  |  |  |  |  |  |  |  |
| None | Reference |  | Reference |  | Reference |  | Reference |  | Reference |  |
| Yes | -0.80  (-4.44 to 2.85) | 0.667 | -0.15  (-4.98 to 4.69) | 0.952 | 3.64  (-1.32 to 8.59) | 0.149 | 0.80 (0.27 to 2.39) | 0.691 | 1.42 (0.51 to 3.92) | 0.504 |
| Baseline PCS | 0.19  (0.09 to 0.28) | <0.001 | 0.23 (0.11 to 0.36) | <0.001 | -0.29  (-0.42 to -0.16) | <0.001 | 0.97  (0.94 to 1.00) | 0.023 | 0.97 (0.94 to 1.00) | 0.023 |
| Baseline MCS | 0.09  (0.02 to 0.16) | 0.014 | 0.33 (0.25 to 0.42) | <0.001 | -0.17  (-0.26 to -0.07) | <0.001 | 0.94 (0.93 to 0.96) | <0.001 | 0.94 (0.91 to 0.96) | <0.001 |
| Baseline DASH | -0.14  (-0.23 to -0.05) | 0.003 | -0.30  (-0.42 to -0.18) | <0.001 | 0.28 (0.15 to 0.40) | <0.001 | 1.04 (1.02 to 1.07) | <0.001 | 1.04 (1.02 to 1.07) | <0.001 |
| Baseline HADS-A |  |  |  |  |  |  |  |  |  |  |
| <8 | Reference |  | Reference |  | Reference |  | Reference |  | Reference |  |
| ≥8 | -2.82  (-5.21 to -0.43) | 0.021 | -7.01  (-10.11 to -3.90) | <0.001 | 4.65  (1.40 to 7.90) | 0.005 | 5.61 (2.99 to 10.53) | <0.001 | 4.03 (2.05 to 7.89) | <0.001 |
| Baseline HADS-D |  |  |  |  |  |  |  |  |  |  |
| <8 | Reference |  | Reference |  | Reference |  | Reference |  | Reference |  |
| ≥8 | -3.83  (-6.65 to -1.01) | 0.008 | -9.28  (-12.93 to -5.64) | <0.001 | 4.50 (0.64 to 8.36) | 0.022 | 6.39 (3.29 to 12.40) | <0.001 | 6.12 (3.02 to 12.39) | <0.001 |
| Baseline symptom severity | -0.30 (-0.67 to 0.06) | 0.101 | -0.40 (-0.88 to 0.08) | 0.104 | 0.47 (-0.02 to 0.96) | 0.062 | 1.03 (0.95 to 1.13) | 0.469 | 1.06 (0.98 to 1.16) | 0.164 |
| Follow-up symptom severity | -0.68  (-0.79 to -0.56) | <0.001 | -0.70  (-0.86 to -0.53) | <0.001 | 1.19 (1.06 to 1.32) | <0.001 | 1.09 (1.05 to 1.13) | <0.001 | 1.10 (1.06 to 1.14) | <0.001 |
| Trajectory groups |  |  |  |  |  |  |  |  |  |  |
| Persistent-low | Reference |  | Reference |  | Reference |  | Reference |  | Reference |  |
| High-stable | -5.28 (-7.80 to -2.76) | <0.001 | -9.24 (-12.63 to -5.85) | <0.001 | 11.97 (8.70 to 15.25) | <0.001 | 3.53 (1.65 to 7.56) | 0.001 | 5.58 (2.48 to 12.58) | <0.001 |
| Low-rapid-progression | -10.47 (-13.14 to -7.80) | <0.001 | -11.12 (-14.72 to -7.52) | <0.001 | 15.31 (11.83 to 18.79) | <0.001 | 6.08 (2.89 to 12.81) | <0.001 | 5.18 (2.21 to 12.16) | <0.001 |

The odds ratios for cTNM stage in the HADS-D univariable analysis were not estimable because of sparse data (complete separation); therefore, only the p-value (from Fisher's exact test) is reported.

Abbreviations: PROs, patient-reported outcomes; OR, odds ratio; 95% CI, 95% confidence interval; BMI, body mass index; cTNM, clinical tumor-node-metastasis stage; pTNM, pathological tumor-node-metastasis stage; BCS, breast-conserving surgery; TAM, tamoxifen; AI, aromatase inhibitor; CCI, Charlson Comorbidity Index; PCS, Physical Component Summary; MCS, Mental Component Summary; DASH, Disabilities of the Arm, Shoulder, and Hand questionnaire; HADS-A, Hospital Anxiety and Depression Scale–anxiety subscale; HADS-D, Hospital Anxiety and Depression Scale–depression subscale.

**Table S3. Sensitivity analysis comparing conventional versus Huber–White robust standard errors for the association between lymphedema symptom trajectories and PROs (n = 332)**

| Outcome | Trajectory group* | Conventional SE  *β*/OR (95% CI) | *p*-value | Robust SE  *β*/OR (95% CI) | *p*-value | Direction consistent |
| --- | --- | --- | --- | --- | --- | --- |
| PCS | High-stable | -2.41 (-5.18 to 0.36) | 0.089 | -2.41 (-5.18 to 0.35) | 0.088 | Yes |
|  | Low-rapid-progression | -9.99 (-12.65 to -7.34) | <0.001 | -9.99 (-13.14 to -6.85) | <0.001 | Yes |
| MCS | High-stable | -5.20 (-8.75 to -1.66) | 0.004 | -5.20 (-8.80 to -1.60) | 0.005 | Yes |
|  | Low-rapid-progression | -9.53 (-12.92 to -6.13) | <0.001 | -9.53 (-13.18 to -5.87) | <0.001 | Yes |
| DASH | High-stable | 8.20 (4.59 to 11.80) | <0.001 | 8.20 (3.82 to 12.57) | <0.001 | Yes |
|  | Low-rapid-progression | 14.44 (10.98 to 17.89) | <0.001 | 14.44 (11.14 to 17.74) | <0.001 | Yes |
| HADS-A‡ | High-stable | 1.93 (0.65 to 5.68) | 0.235 | 1.93 (0.67 to 5.55) | 0.225 | Yes |
|  | Low-rapid-progression | 4.75 (1.96 to 11.48) | <0.001 | 4.75 (1.85 to 12.17) | <0.001 | Yes |
| HADS-D‡ | High-stable | 4.35 (1.44 to 13.18) | 0.009 | 4.35 (1.34 to 14.14) | 0.015 | Yes |
|  | Low-rapid-progression | 4.21 (1.57 to 11.27) | 0.004 | 4.21 (1.51 to 11.72) | 0.006 | Yes |

*Reference group: persistent-low trajectory.

Adjusted for age, educational level, marital status, employment status, BMI, follow-up time point, ALND affected dominant arm, chemotherapy, taxane-based chemotherapy, radiotherapy, and baseline PCS, MCS, HADS, and DASH scores, and weight change from baseline (Model III covariates). ‡Logistic regression; estimates are odds ratios (OR) for HADS-A/HADS-D ≥ 8; PCS, MCS, and DASH are linear regression coefficients (β). Conventional standard errors were obtained from the standard model fit; Huber–White robust standard errors were estimated using the sandwich variance estimator (HC1).

Abbreviations: PCS, Physical Component Summary; MCS, Mental Component Summary; DASH, Disabilities of the Arm, Shoulder, and Hand questionnaire; HADS-A, Hospital Anxiety and Depression Scale–anxiety subscale; HADS-D, Hospital Anxiety and Depression Scale–depression subscale; β, linear regression coefficient; OR, odds ratio; CI, confidence interval.

**Table S4. Sensitivity analysis: association between lymphedema symptom trajectories and PROs restricted to participants with complete data at all three time points (n = 117), compared with the primary analysis (n = 332)**

| Outcome | Trajectory group | Primary analysis (n = 332)* | | Completers only (n = 117)† | | Direction consistent |
| --- | --- | --- | --- | --- | --- | --- |
|  |  | *β* / OR (95% CI) | *p*-value | *β* / OR (95% CI) | *p*-value |  |
| PCS | High-stable | -2.41 (-5.18 to 0.36) | 0.089 | -4.16 (-8.74 to 0.42) | 0.078 | Yes |
|  | Low-rapid-progression | -9.99 (-12.65 to -7.34) | <0.001 | -10.05 (-13.32 to -6.79) | <0.001 | Yes |
| MCS | High-stable | -5.20 (-8.75 to -1.66) | 0.004 | -5.04 (-12.34 to 2.27) | 0.180 | Yes |
|  | Low-rapid-progression | -9.53 (-12.92 to -6.13) | <0.001 | -7.87 (-13.08 to -2.66) | 0.004 | Yes |
| DASH | High-stable | 8.20 (4.59 to 11.80) | <0.001 | 6.73 (-0.02 to 13.43) | 0.052 | Yes |
|  | Low-rapid-progression | 14.44 (10.98 to 17.89) | <0.001 | 12.20 (7.42 to 16.99) | <0.001 | Yes |
| HADS-A§  (continuous) | High-stable | – | – | 0.91 (-1.09 to 2.91) | 0.375 | Yes |
|  | Low-rapid-progression | – | – | 2.28 (0.86 to 3.71) | 0.002 | Yes |
| HADS-D§  (continuous) | High-stable | – | – | 1.32 (-0.70 to 3.35) | 0.203 | Yes |
|  | Low-rapid-progression | – | – | 1.44 (0.00 to 2.88) | 0.054 | Yes |
| HADS-A‡  (binary ≥8) | High-stable | 1.93 (0.65 to 5.68) | 0.235 | 1.27 (0.03 to 49.05) | 0.897 | Yes |
|  | Low-rapid-progression | 4.75 (1.96 to 11.48) | 0.001 | 4.89 (0.60 to 39.80) | 0.138 | Yes |
| HADS-D‡  (binary ≥8) | High-stable | 4.35 (1.44 to 13.18) | 0.009 | 22.60 (0.48 to 1057.08) | 0.112 | Yes |
|  | Low-rapid-progression | 4.21 (1.57 to 11.27) | 0.004 | 1.54 (0.14 to 16.90) | 0.722 | Yes |

*Reference group: persistent-low trajectory. Adjusted for age, educational level, marital status, employment status, BMI, follow-up time point, ALND affected dominant arm, chemotherapy, taxane-based chemotherapy, radiotherapy, baseline PRO scores (PCS, MCS, HADS, DASH), and weight change from baseline (Model III covariates). Outcome = last available follow-up PRO assessment.

†Same covariates as the primary analysis except that follow-up time point was not included, because all outcomes were assessed at 24 months; weight change from baseline (relative to the 24-month assessment) was retained. Outcome = 24-month PRO assessment.

‡Logistic regression; estimates are odds ratios (OR) for possible anxiety/depression symptoms (HADS-A/D ≥ 8).

§Linear regression; estimates are β coefficients for continuous HADS scores (0–21). These models were added because the small number of events in the reduced sample yielded very wide confidence intervals for the binary outcomes; they are presented as supplementary evidence that the direction of the trajectory–HADS associations is preserved.

–, Not estimated in the primary analysis.

"Direction consistent" refers to the direction of effect relative to the primary analysis, not to statistical significance in the reduced sample.

Abbreviations: PCS, Physical Component Summary; MCS, Mental Component Summary; DASH, Disabilities of the Arm, Shoulder, and Hand questionnaire; HADS-A, Hospital Anxiety and Depression Scale–anxiety subscale; HADS-D, Hospital Anxiety and Depression Scale–depression subscale; β, linear regression coefficient; OR, odds ratio; CI, confidence interval.

**Table S5. Sensitivity analysis: association of lymphedema-specific symptom trajectories (12-item subset) with PROs**

Panel A. Fit statistics for the lymphedema-specific symptom trajectories (12-item subset)†

| Fit statistic | Number of classes | | | |
| --- | --- | --- | --- | --- |
|  | 2 | 3 | 4 | 5 |
| BIC＊ | 1803.09 | 1201.67 | 1143.05 | 1326.67 |
| AIC＊ | 1746.01 | 1125.57 | 1047.92 | 1212.51 |
| SABIC＊ | 1755.51 | 1138.23 | 1063.75 | 1231.51 |
| Entropy^ | 1.00 | 0.89 | 0.91 | 1.00 |
| Class proportion＃ | Class 1, 83.1% | Class 1, 65.1% | Class 1, 19.6% | Class 1, 0.0% |
|  | Class 2, 16.9% | Class 2, 15.1% | Class 2, 65.1% | Class 2, 82.8% |
|  |  | Class 3, 19.8% | Class 3, 15.1% | Class 3, 8.7% |
|  |  |  | Class 4, 0.2% | Class 4, 7.8% |
|  |  |  |  | Class 5, 0.7% |
| PP☨ | Class 1, 1.00 | Class 1, 0.96 | Class 1, 0.96 | Class 1, 0.00 |
|  | Class 2, 1.00 | Class 2, 1.00 | Class 2, 0.95 | Class 2, 1.00 |
|  |  | Class 3, 1.00 | Class 3, 1.00 | Class 3, 1.00 |
|  |  |  | Class 4, 1.00 | Class 4, 0.97 |
|  |  |  |  | Class 5, 1.00 |

†Three classes were retained for consistency with the primary analysis. ＊Lower values indicate better model fit. ^The value ranges from 0 to 1, and higher values indicate better model fit. ＃Each class comprises at least 5% of the total sample. ☨Higher values indicate better classification (preferably > 0.7 per class).

Panel B. Association between lymphedema-specific (12-item) symptom trajectories and PROs, compared with the primary analysis (full 24-item BCLE-SEI)

| Outcome | Trajectory group* | Primary (24-item)  *β*/OR (95% CI) | *p-value* | Subset (12-item)  *β*/OR (95% CI) | *p-value* |
| --- | --- | --- | --- | --- | --- |
| PCS | High-stable | -2.41 (-5.18 to 0.36) | 0.089 | -3.48 (-6.55 to -0.42) | 0.027 |
|  | Low-rapid-progression | -9.99 (-12.65 to -7.34) | <0.001 | -8.40 (-10.92 to -5.88) | <0.001 |
| MCS | High-stable | -5.20 (-8.75 to -1.66) | 0.004 | -7.48 (-11.37 to -3.58) | <0.001 |
|  | Low-rapid-progression | -9.53 (-12.92 to -6.13) | <0.001 | -7.24 (-10.44 to -4.04) | <0.001 |
| DASH | High-stable | 8.20 (4.59 to 11.80) | <0.001 | 9.10 (5.09 to 13.11) | <0.001 |
|  | Low-rapid-progression | 14.44 (10.98 to 17.89) | <0.001 | 12.08 (8.79 to 15.37) | <0.001 |
| HADS-A‡ | High-stable | 1.93 (0.65 to 5.68) | 0.235 | 2.39 (0.78 to 7.27) | 0.126 |
|  | Low-rapid-progression | 4.75 (1.96 to 11.48) | 0.001 | 3.62 (1.52 to 8.59) | 0.004 |
| HADS-D‡ | High-stable | 4.35 (1.44 to 13.18) | 0.009 | 6.37 (1.99 to 20.35) | 0.002 |
|  | Low-rapid-progression | 4.21 (1.57 to 11.27) | 0.004 | 4.78 (1.81 to 12.62) | 0.002 |

The 12-item subset comprises the swelling or edema-specific BCLE-SEI symptoms (arm/breast/chest-wall swelling, stiffness, tightness, heaviness, fibrosis, firmness, tenderness, hotness, redness, blistering), excluding functional items (limited movement, pain, numbness, weakness, fatigue).

*Reference group: persistent-low trajectory. Adjusted for age, educational level, marital status, employment status, BMI, follow-up time point, ALND affected dominant arm, chemotherapy, taxane-based chemotherapy, radiotherapy, baseline PRO scores, and weight change from baseline (Model III covariates).

‡Logistic regression; estimates are odds ratios (OR) for HADS-A/HADS-D ≥ 8; PCS, MCS, and DASH are linear regression coefficients (*β*).

Panel C. Trajectory patterns of the 12-item lymphedema-specific symptom subset

Three distinct symptom trajectories were identified from the 12-item swelling or edema-specific subset, consistent with the primary analysis (Fig. S1):

**Persistent-low** trajectory **(65.1%)**: symptom scores remained low throughout the 24-month follow-up.

**High-stable** trajectory **(15.1%)**: symptom scores were elevated from baseline and showed little further change.

**Low-rapid-progression** trajectory **(19.8%)**: symptoms started low but increased steeply after surgery, converging with the high-stable trajectory in the later follow-up period.

Abbreviations: AIC, Akaike's information criterion; BIC, Bayesian information criterion; PP, posterior probability; SABIC, sample-size-adjusted BIC; PCS, Physical Component Summary; MCS, Mental Component Summary; DASH, Disabilities of the Arm, Shoulder, and Hand questionnaire; HADS-A, Hospital Anxiety and Depression Scale–anxiety subscale; HADS-D, Hospital Anxiety and Depression Scale–depression subscale; OR, odds ratio; CI, confidence interval; *β*, regression coefficient

**Table S6. E-value analysis for unmeasured confounding (low-rapid-progression vs. persistent-low trajectory, Model III)**

| Outcome | Effect estimate (95% CI) | E-value (point estimate) | E-value (CI lower limit) |
| --- | --- | --- | --- |
| PCS‡ | β = -9.99 (-12.65 to -7.34) | 4.5 | 3.4 |
| MCS‡ | β = -9.53 (-12.92 to -6.13) | 3.3 | 2.4 |
| DASH‡ | β = 14.44 (10.98 to 17.89) | 4.8 | 3.6 |
| HADS-A§ | OR = 4.75 (1.96 to 11.48) | 9.0 | 3.3 |
| HADS-D§ | OR = 4.21 (1.57 to 11.27) | 7.9 | 2.5 |

The E-value is the minimum strength of association, on the risk-ratio scale, that an unmeasured confounder would need to have with both the trajectory group and the outcome (conditional on the measured covariates) to fully explain away the observed association. "E-value (point)" uses the point estimate; "E-value (CI lower limit)" uses the confidence-interval limit closest to the null, which is the more conservative bound. Computed using the EValue R package [31].

‡Continuous outcomes: E-value computed from the standardized mean difference (β divided by the outcome SD; PCS SD = 9.8, MCS SD = 13.0, DASH SD = 13.4).

§Binary outcomes: E-value computed from the odds ratio.

Abbreviations: PCS, Physical Component Summary; MCS, Mental Component Summary; DASH, Disabilities of the Arm, Shoulder, and Hand questionnaire; HADS-A, Hospital Anxiety and Depression Scale–anxiety subscale; HADS-D, Hospital Anxiety and Depression Scale–depression subscale; β, linear regression coefficient; OR, odds ratio; CI, confidence interval; SD, standard deviation.

**Table S7. Prevalence and severity of lymphedema-related symptoms (n = 332)**

| No. | Symptoms | n (%) | Severity | |
| --- | --- | --- | --- | --- |
|  |  |  | Mean (SD) | Median (IQR) |
| L1 | Limited shoulder movement | 122 (36.7) | 1.34 (0.58) | 1.00 (1.00, 2.00) |
| L2 | Limited elbow movement | 52 (15.7) | 1.38 (0.66) | 1.00 (1.00, 2.00) |
| L3 | Limited wrist movement | 75 (22.6) | 1.32 (0.57) | 1.00 (1.00, 2.00) |
| L4 | Limited fingers movement | 75 (22.6) | 1.32 (0.57) | 1.00 (1.00, 2.00) |
| L5 | Limited arm movement | 103 (31.0) | 1.35 (0.57) | 1.00 (1.00, 2.00) |
| S1 | Arm swelling | 146 (44.0) | 1.18 (0.45) | 1.00 (1.00, 1.00) |
| S2 | Breast swelling | 51 (15.4) | 1.14 (0.35) | 1.00 (1.00, 1.00) |
| S3 | Chest-wall swelling | 57 (17.2) | 1.09 (0.29) | 1.00 (1.00, 1.00) |
| S4 | Stiffness | 121 (36.4) | 1.15 (0.40) | 1.00 (1.00, 1.00) |
| S5 | Tightness | 112 (33.7) | 1.18 (0.45) | 1.00 (1.00, 1.00) |
| S6 | Heaviness | 104 (31.3) | 1.22 (0.48) | 1.00 (1.00, 1.00) |
| S7 | Fibrosis (toughness or thickness of skin) | 55 (16.6) | 1.18 (0.51) | 1.00 (1.00, 1.00) |
| S8 | Firmness | 85 (25.6) | 1.21 (0.58) | 1.00 (1.00, 1.00) |
| S9 | Tenderness | 84 (25.3) | 1.13 (0.37) | 1.00 (1.00, 1.00) |
| S10 | Hotness | 29 (8.7) | 1.10 (0.41) | 1.00 (1.00, 1.00) |
| S11 | Redness | 21 (6.3) | 1.14 (0.48) | 1.00 (1.00, 1.00) |
| S12 | Blistering | 4 (1.2) | 1.50 (1.00) | 1.00 (1.00, 2.00) |
| S13 | Pain/aching/soreness | 111 (33.4) | 1.14 (0.42) | 1.00 (1.00, 1.00) |
| S14 | Numbness | 125 (37.7) | 1.10 (0.35) | 1.00 (1.00, 1.00) |
| S15 | Burning | 19 (5.7) | 1.11 (0.46) | 1.00 (1.00, 1.00) |
| S16 | Stabbing | 45 (13.6) | 1.07 (0.33) | 1.00 (1.00, 1.00) |
| S17 | Tingling | 48 (14.5) | 1.08 (0.35) | 1.00 (1.00, 1.00) |
| S18 | Fatigue | 117 (35.2) | 1.16 (0.45) | 1.00 (1.00, 1.00) |
| S19 | Weakness | 119 (35.8) | 1.14 (0.44) | 1.00 (1.00, 1.00) |

**Table S8. Mixed-effects model for repeated measures of PROs**

| Variables | PCS | | MCS | | DASH | | HADS-A | | HADS-D | |
| --- | --- | --- | --- | --- | --- | --- | --- | --- | --- | --- |
|  | *β* (95% CI) | *p*-value | *β* (95% CI) | *p*-value | *β* (95% CI) | *p*-value | OR (95% CI) | *p*-value | OR (95% CI) | *p*-value |
| Intercept | 49.41 (48.12 to 50.71) | <0.001 | 45.66 (43.86 to 47.45) | <0.001 | 3.99 (2.51 to 5.48) | <0.001 | 0.11 (0.06 to 0.21) | <0.001 | 0.02 (0.00 to 0.11) | <0.001 |
| High-stable | -9.92 (-12.61 to -7.23) | <0.001 | -7.90 (-11.64 to -4.16) | <0.001 | 10.90 (7.81 to 14.00) | <0.001 | 3.77 (1.45 to 9.82) | 0.007 | 6.40 (1.78 to 23.00) | 0.004 |
| Low-rapid-progression | -4.02 (-6.88 to -1.16) | 0.006 | -4.74 (-8.71 to -0.78) | 0.019 | 2.72 (-0.57 to 6.00) | 0.105 | 4.03 (1.46 to 11.13) | 0.007 | 2.39 (0.59 to 9.79) | 0.225 |
| 12 months | -1.64 (-3.27 to -0.01) | 0.049 | 6.63 (4.68 to 8.58) | <0.001 | 4.64 (2.83 to 6.46) | <0.001 | 0.26 (0.13 to 0.52) | <0.001 | 0.34 (0.13 to 0.84) | 0.020 |
| 24 months | 0.56 (-1.82 to 2.94) | 0.644 | 8.28 (5.36 to 11.20) | <0.001 | 2.99 (0.33 to 5.66) | 0.028 | 0.31 (0.11 to 0.87) | 0.026 | 0.57 (0.18 to 1.83) | 0.342 |
| High-stable × 12 months | 4.45 (1.05 to 7.84) | 0.011 | -1.69 (-5.75 to 2.37) | 0.416 | 1.45 (-2.32 to 5.22) | 0.451 | 1.53 (0.47 to 4.97) | 0.483 | 1.66 (0.41 to 6.67) | 0.474 |
| Low-rapid-progression × 12 months | -5.61 (-9.23 to -1.98) | 0.003 | -6.90 (-11.23 to -2.56) | 0.002 | 12.60 (8.57 to 16.63) | <0.001 | 3.60 (1.07 to 12.10) | 0.038 | 7.69 (1.47 to 40.34) | 0.016 |
| High-stable × 24 months | 4.61 (-0.78 to 10.00) | 0.094 | 1.13 (-5.51 to 7.77) | 0.739 | -0.95 (-6.99 to 5.09) | 0.758 | 0.52 (0.06 to 4.34) | 0.547 | 0.87 (0.09 to 8.15) | 0.900 |
| Low-rapid-progression × 24 months | -6.97 (-11.64 to -2.31) | 0.004 | -4.86 (-10.56 to 0.84) | 0.095 | 11.41 (6.19 to 16.62) | <0.001 | 2.80 (0.55 to 14.16) | 0.213 | 1.54 (0.19 to 12.37) | 0.685 |

The mixed-effects model was adjusted for weight change from baseline as a time-varying covariate. Continuous outcomes (PCS, MCS, and DASH) were fitted by restricted maximum likelihood. HADS-A and HADS-D were analyzed as binary outcomes (score ≥ 8, indicating possible anxiety or depression symptoms) using generalized linear mixed-effects models with a logit link, fitted by adaptive Gauss–Hermite quadrature; estimates for these outcomes are odds ratios, whereas those for PCS, MCS, and DASH are regression coefficients (*β*).

Abbreviations: PROs, patient-reported outcomes; OR, odds ratio; 95% CI, 95% confidence interval; PCS, Physical Component Summary; MCS, Mental Component Summary; HADS-A, Hospital Anxiety and Depression Scale–anxiety subscale; HADS-D, Hospital Anxiety and Depression Scale–depression subscale; DASH, Disabilities of the Arm, Shoulder, and Hand questionnaire.

**Table S9. Association between the lymphedema symptom trajectories and PROs across subgroups**

| Variable | PCS | | MCS | | HADS-A | | HADS-D | | DASH | |
| --- | --- | --- | --- | --- | --- | --- | --- | --- | --- | --- |
|  | *β* (95% CI) | *p*-value | *β* (95% CI) | *p*-value | *β* (95% CI) | *p*-value | *β* (95% CI) | *p*-value | *β* (95% CI) | *p*-value |
| **Age, years** |  | 0.838 |  | 0.800 |  | 0.521 |  | 0.521 |  | 0.105 |
| **<50** |  |  |  |  |  |  |  |  |  |  |
| Persistent-low | 0 (Ref) |  | 0 (Ref) |  | 0 (Ref) |  | 0 (Ref) |  | 0 (Ref) |  |
| High-stable | -1.72 (-4.85 to 1.41) | 0.283 | -5.28 (-9.61 to -0.96) | 0.018 | 0.62 (-0.46 to 1.71) | 0.263 | 0.59 (-0.54 to 1.71) | 0.308 | 5.87 (2.19 to 9.54) | 0.002 |
| Low-rapid-progression | -10.80 (-13.97 to -7.63) | <0.001 | -9.91 (-14.29 to -5.54) | <0.001 | 2.01 (0.91 to 3.11) | <0.001 | 2.00 (0.86 to 3.13) | 0.001 | 11.30 (7.57 to 15.02) | <0.001 |
| p for trend |  | <0.001 |  | <0.001 |  | <0.001 |  | 0.001 |  | <0.001 |
| **≥50** |  |  |  |  |  |  |  |  |  |  |
| Persistent-low | 0 (Ref) |  | 0 (Ref) |  | 0 (Ref) |  | 0 (Ref) |  | 0 (Ref) |  |
| High-stable | -4.23 (-10.26 to 1.81) | 0.173 | -3.26 (-10.33 to 3.82) | 0.369 | 0.57 (-1.29 to 2.43) | 0.546 | 1.88 (0.11 to 3.66) | 0.040 | 13.11 (5.12 to 21.11) | 0.002 |
| Low-rapid-progression | -10.43 (-15.50 to -5.37) | <0.001 | -9.28 (-15.22 to -3.33) | 0.003 | 2.72 (1.16 to 4.28) | 0.001 | 2.32 (0.83 to 3.81) | 0.003 | 19.20 (12.48 to 25.92) | <0.001 |
| p for trend |  | <0.001 |  | 0.003 |  | 0.001 |  | 0.001 |  | <0.001 |
| **Educational level, years** |  | 0.612 |  | 0.470 |  | 0.834 |  | 0.899 |  | 0.209 |
| **<12** |  |  |  |  |  |  |  |  |  |  |
| Persistent-low | 0 (Ref) |  | 0 (Ref) |  | 0 (Ref) |  | 0 (Ref) |  | 0 (Ref) |  |
| High-stable | -3.04 (-8.53 to 2.45) | 0.280 | -4.13 (-10.83 to 2.57) | 0.229 | 0.85 (-0.92 to 2.62) | 0.347 | 0.77 (-0.84 to 2.38) | 0.349 | 7.11 (-0.53 to 14.75) | 0.071 |
| Low-rapid-progression | -10.82 (-16.08 to -5.57) | <0.001 | -9.19 (-15.60 to -2.78) | 0.006 | 2.09 (0.40 to 3.79) | 0.017 | 2.38 (0.85 to 3.92) | 0.003 | 14.92 (7.60 to 22.23) | <0.001 |
| p for trend |  | <0.001 |  | 0.004 |  | 0.007 |  | 0.003 |  | <0.001 |
| **≥12** |  |  |  |  |  |  |  |  |  |  |
| Persistent-low | 0 (Ref) |  | 0 (Ref) |  | 0 (Ref) |  | 0 (Ref) |  | 0 (Ref) |  |
| High-stable | -2.60 (-5.93 to 0.73) | 0.127 | -7.04 (-11.46 to -2.62) | 0.002 | 1.11 (0.02 to 2.20) | 0.048 | 1.42 (0.25 to 2.59) | 0.019 | 10.12 (6.12 to 14.12) | <0.001 |
| Low-rapid-progression | -9.41 (-12.52 to -6.31) | <0.001 | -9.24 (-13.36 to -5.12) | <0.001 | 2.32 (1.30 to 3.34) | <0.001 | 2.01 (0.92 to 3.11) | <0.001 | 13.65 (9.92 to 17.38) | <0.001 |
| p for trend |  | <0.001 |  | <0.001 |  | <0.001 |  | <0.001 |  | <0.001 |
| **Marital status** |  | 0.794 |  | 0.219 |  | 0.683 |  | 0.927 |  | 0.625 |
| **Married or living with a partner** |  |  |  |  |  |  |  |  |  |  |
| Persistent-low | 0 (Ref) |  | 0 (Ref) |  | 0 (Ref) |  | 0 (Ref) |  | 0 (Ref) |  |
| High-stable | -3.17 (-6.20 to -0.14) | 0.042 | -4.66 (-8.64 to -0.67) | 0.023 | 0.68 (-0.36 to 1.71) | 0.203 | 1.13 (0.06 to 2.19) | 0.039 | 7.70 (3.60 to 11.81) | <0.001 |
| Low-rapid-progression | -9.85 (-12.62 to -7.08) | <0.001 | -10.32 (-13.97 to -6.68) | <0.001 | 2.46 (1.51 to 3.41) | <0.001 | 2.19 (1.22 to 3.16) | <0.001 | 14.07 (10.32 to 17.83) | <0.001 |
| p for trend |  | <0.001 |  | <0.001 |  | <0.001 |  | <0.001 |  | <0.001 |
| **Living alone** |  |  |  |  |  |  |  |  |  |  |
| Persistent-low | 0 (Ref) |  | 0 (Ref) |  | 0 (Ref) |  | 0 (Ref) |  | 0 (Ref) |  |
| High-stable | 3.53 (-7.75 to 14.81) | 0.547 | -7.53 (-17.03 to 1.97) | 0.137 | 1.42 (-0.46 to 3.31) | 0.156 | 0.30 (-1.45 to 2.06) | 0.739 | 10.65 (1.51 to 19.80) | 0.034 |
| Low-rapid-progression | -13.85 (-26.27 to -1.43) | 0.042 | 5.61 (-4.85 to 16.07) | 0.306 | 1.44 (-0.64 to 3.52) | 0.190 | 1.78 (-0.15 to 3.71) | 0.086 | 16.17 (6.10 to 26.25) | 0.005 |
| p for trend |  | 0.116 |  | 0.683 |  | 0.111 |  | 0.101 |  | 0.002 |
| **Employment status** |  | 0.963 |  | 0.414 |  | 0.638 |  | 0.360 |  | 0.533 |
| **Employed** |  |  |  |  |  |  |  |  |  |  |
| Persistent-low | 0 (Ref) |  | 0 (Ref) |  | 0 (Ref) |  | 0 (Ref) |  | 0 (Ref) |  |
| High-stable | -2.28 (-5.57 to 1.01) | 0.176 | -6.74 (-11.05 to -2.43) | 0.003 | 0.74 (-0.42 to 1.89) | 0.213 | 1.20 (0.03 to 2.38) | 0.062 | 7.91 (3.87 to 11.95) | <0.001 |
| Low-rapid-progression | -10.26 (-13.37 to -7.16) | <0.001 | -11.13 (-15.20 to -7.06) | <0.001 | 2.59 (1.50 to 3.68) | <0.001 | 2.67 (1.56 to 3.78) | <0.001 | 12.72 (8.91 to 16.54) | <0.001 |
| p for trend |  | <0.001 |  | <0.001 |  | <0.001 |  | <0.001 |  | <0.001 |
| **Unemployed or retired** |  |  |  |  |  |  |  |  |  |  |
| Persistent-low | 0 (Ref) |  | 0 (Ref) |  | 0 (Ref) |  | 0 (Ref) |  | 0 (Ref) |  |
| High-stable | -3.33 (-8.37 to 1.71) | 0.197 | -4.38 (-10.87 to 2.12) | 0.189 | 0.98 (-0.62 to 2.58) | 0.234 | 1.12 (-0.44 to 2.68) | 0.163 | 10.05 (3.14 to 16.96) | 0.005 |
| Low-rapid-progression | -10.84 (-15.95 to -5.74) | 0.001 | -7.01 (-13.60 to -0.43) | 0.039 | 2.10 (0.48 to 3.72) | 0.012 | 1.43 (-0.15 to 3.02) | 0.078 | 18.14 (11.14 to 25.15) | <0.001 |
| p for trend |  | <0.001 |  | 0.025 |  | 0.009 |  | 0.045 |  | <0.001 |
| **BMI, kg/m^2^** |  | 0.972 |  | 0.320 |  | 0.110 |  | 0.159 |  | 0.906 |
| **<25** |  |  |  |  |  |  |  |  |  |  |
| Persistent-low | 0 (Ref) |  | 0 (Ref) |  | 0 (Ref) |  | 0 (Ref) |  | 0 (Ref) |  |
| High-stable | -1.98 (-5.49 to 1.53) | 0.270 | -7.12 (-11.50 to -2.74) | 0.002 | 1.38 (0.30 to 2.47) | 0.014 | 1.69 (0.58 to 2.80) | 0.003 | 8.76 (4.31 to 13.21) | <0.001 |
| Low-rapid-progression | -9.78 (-13.05 to -6.51) | <0.001 | -9.81 (-13.89 to -5.72) | <0.001 | 2.00 (0.98 to 3.01) | <0.001 | 2.02 (0.98 to 3.05) | <0.001 | 13.95 (9.81 to 18.10) | <0.001 |
| p for trend |  | <0.001 |  | <0.001 |  | <0.001 |  | <0.001 |  | <0.001 |
| **≥25** |  |  |  |  |  |  |  |  |  |  |
| Persistent-low | 0 (Ref) |  | 0 (Ref) |  | 0 (Ref) |  | 0 (Ref) |  | 0 (Ref) |  |
| High-stable | -4.29 (-8.98 to -0.40) | 0.075 | -3.19 (-9.28 to 2.91) | 0.308 | 0.31 (-1.26 to 1.87) | 0.702 | 0.35 (-1.29 to 1.99) | 0.675 | 9.58 (3.20 to 15.97) | 0.004 |
| Low-rapid-progression | -10.30 (-14.88 to -5.71) | <0.001 | -9.55 (-15.51 to -3.58) | 0.002 | 3.10 (1.57 to 4.62) | <0.001 | 2.51 (0.90 to 4.12) | 0.003 | 14.82 (8.58 to 21.06) | <0.001 |
| p for trend |  | <0.001 |  | 0.002 |  | <0.001 |  | 0.005 |  | <0.001 |
| ALND affected dominant arm |  | 0.385 |  | 0.398 |  | 0.606 |  | 0.986 |  | 0.101 |
| **None** |  |  |  |  |  |  |  |  |  |  |
| Persistent-low | 0 (Ref) |  | 0 (Ref) |  | 0 (Ref) |  | 0 (Ref) |  | 0 (Ref) |  |
| High-stable | -2.11 (-6.43 to 2.20) | 0.339 | -4.69 (-10.02 to 0.63) | 0.086 | 0.81 (-0.58 to 2.20) | 0.254 | 0.86 (-0.48 to 2.21) | 0.209 | 4.03 (-0.98 to 9.04) | 0.117 |
| Low-rapid-progression | -8.04 (-12.10 to -3.99) | <0.001 | -8.41 (-13.42 to -3.41) | 0.001 | 2.08 (0.77 to 3.39) | 0.002 | 2.37 (1.11 to 3.63) | <0.001 | 11.85 (7.13 to 16.56) | <0.001 |
| p for trend |  | <0.001 |  | 0.001 |  | 0.002 |  | <0.001 |  | <0.001 |
| **Yes** |  |  |  |  |  |  |  |  |  |  |
| Persistent-low | 0 (Ref) |  | 0 (Ref) |  | 0 (Ref) |  | 0 (Ref) |  | 0 (Ref) |  |
| High-stable | -4.16 (-8.03 to -0.28) | 0.037 | -3.98 (-8.95 to 0.99) | 0.118 | 0.48 (-0.82 to 1.78) | 0.468 | 1.05 (-0.35 to 2.44) | 0.143 | 12.67 (7.03 to 18.31) | <0.001 |
| Low-rapid-progression | -12.37 (-16.05 to -8.70) | <0.001 | -10.88 (-15.59 to -6.16) | <0.001 | 2.69 (1.46 to 3.92) | <0.001 | 2.26 (0.94 to 3.58) | 0.001 | 17.52 (12.17 to 22.86) | <0.001 |
| p for trend |  | <0.001 |  | <0.001 |  | <0.001 |  | 0.001 |  | <0.001 |
| **Taxane-based chemotherapy** |  | 0.097 |  | 0.065 |  | 0.131 |  | 0.116 |  | 0.262 |
| **None** |  |  |  |  |  |  |  |  |  |  |
| Persistent-low | 0 (Ref) |  | 0 (Ref) |  | 0 (Ref) |  | 0 (Ref) |  | 0 (Ref) |  |
| High-stable | -1.71 (-9.10 to 5.68) | 0.652 | 5.05 (-3.00 to 13.09) | 0.225 | -1.92 (-4.25 to 0.41) | 0.113 | -1.66 (-3.43 to 0.12) | 0.074 | 0.57 (-6.44 to 7.58) | 0.874 |
| Low-rapid-progression | -3.75 (-9.74 to 2.23) | 0.225 | -10.94 (-17.45 to -4.43) | 0.002 | 1.85 (-0.04 to 3.74) | 0.060 | 1.70 (0.26 to 3.14) | 0.025 | 13.46 (7.79 to 19.13) | <0.001 |
| p for trend |  | 0.211 |  | 0.011 |  | 0.184 |  | 0.111 |  | <0.001 |
| **Yes** |  |  |  |  |  |  |  |  |  |  |
| Persistent-low | 0 (Ref) |  | 0 (Ref) |  | 0 (Ref) |  | 0 (Ref) |  | 0 (Ref) |  |
| High-stable | -2.53 (-5.57 to 0.51) | 0.104 | -6.91 (-10.75 to -3.08) | <0.001 | 1.15 (0.17 to 2.12) | 0.022 | 1.62 (0.61 to 2.63) | 0.002 | 9.58 (5.44 to 13.72) | <0.001 |
| Low-rapid-progression | -11.66 (-14.64 to -8.68) | <0.001 | -9.62 (-13.38 to -5.85) | <0.001 | 2.68 (1.72 to 3.64) | <0.001 | 2.45 (1.46 to 3.44) | <0.001 | 15.20 (11.13 to 19.26) | <0.001 |
| p for trend |  | <0.001 |  | <0.001 |  | <0.001 |  | <0.001 |  | <0.001 |
| **Radiotherapy** |  | 0.396 |  | 0.277 |  | 0.250 |  | 0.036 |  | 0.204 |
| **None** |  |  |  |  |  |  |  |  |  |  |
| Persistent-low | 0 (Ref) |  | 0 (Ref) |  | 0 (Ref) |  | 0 (Ref) |  | 0 (Ref) |  |
| High-stable | -7.27 (-16.77 to 2.23) | 0.142 | -7.08 (-17.02 to 2.85) | 0.170 | 0.40 (-2.69 to 3.49) | 0.803 | -1.52 (-4.20 to 1.16) | 0.274 | 9.10 (-4.14 to 22.34) | 0.185 |
| Low-rapid-progression | -5.53 (-12.70 to 1.64) | 0.138 | -15.31 (-22.81 to -7.82) | <0.001 | 2.79 (0.46 to 5.12) | 0.024 | 1.64 (-0.38 to 3.67) | 0.119 | 17.05 (7.06 to 27.03) | 0.002 |
| p for trend |  | 0.087 |  | <0.001 |  | 0.028 |  | 0.226 |  | 0.001 |
| **Yes** |  |  |  |  |  |  |  |  |  |  |
| Persistent-low | 0 (Ref) |  | 0 (Ref) |  | 0 (Ref) |  | 0 (Ref) |  | 0 (Ref) |  |
| High-stable | -2.68 (-5.67 to 0.32) | 0.081 | -6.35 (-10.17 to -2.52) | 0.001 | 1.09 (0.10 to 2.07) | 0.031 | 1.68 (0.68 to 2.68) | 0.001 | 9.01 (5.16 to 12.86) | <0.001 |
| Low-rapid-progression | -10.67 (-13.53 to -7.81) | <0.001 | -9.80 (-13.45 to -6.14) | <0.001 | 2.47 (1.53 to 3.41) | <0.001 | 2.46 (1.51 to 3.41) | <0.001 | 14.22 (10.54 to 17.89) | <0.001 |
| p for trend |  | <0.001 |  | <0.001 |  | <0.001 |  | <0.001 |  | <0.001 |

In each stratified analysis, models were adjusted for all covariates other than the stratification variable (age, educational level, marital status, employment status, BMI, follow-up time point, ALND affected dominant arm, chemotherapy, taxane-based chemotherapy, radiotherapy, and baseline PCS, MCS, HADS, and DASH scores, and weight change from baseline). HADS-A and HADS-D were analyzed as continuous scores (0–21) in the subgroup analyses; all effect estimates are reported as regression coefficients (*β*) with 95% confidence intervals (CIs).

Subgroup analyses were exploratory and were not corrected for multiple comparisons; the radiotherapy × HADS-D interaction (*p* = 0.036) should therefore be interpreted with caution.

Abbreviations: 95% CI, 95% confidence interval; PCS, Physical Component Summary; MCS, Mental Component Summary; DASH, Disabilities of the Arm, Shoulder, and Hand questionnaire; HADS-A, Hospital Anxiety and Depression Scale–anxiety subscale; HADS-D, Hospital Anxiety and Depression Scale–depression subscale; BMI, body mass index.

**Table S10. Baseline characteristics of participants in the analytic cohort (n = 332) versus those excluded (n = 51)**

| Variables | Total (n = 383) | Excluded (n = 51) | Included (n = 332) | *p*-value |
| --- | --- | --- | --- | --- |
| Age (years), Mean (SD) | 48.2 (9.6) | 52.5 (9.6) | 47.6 (9.4) | <0.001 |
| Educational level (years), n (%) |  |  |  | <0.001 |
| <12 | 164 (42.8) | 35 (68.6) | 129 (38.9) |  |
| ≥12 | 219 (57.2) | 16 (31.4) | 203 (61.1) |  |
| Marital status, n (%) |  |  |  | 0.381 |
| Married or living with a partner | 339 (88.5) | 47 (92.2) | 292 (88.0) |  |
| Living alone | 44 (11.5) | 4 (7.8) | 40 (12.0) |  |
| Employment status, n (%) |  |  |  | <0.001 |
| Employed | 208 (54.3) | 14 (27.5) | 194 (58.4) |  |
| Unemployed | 61 (15.9) | 14 (27.5) | 47 (14.2) |  |
| Retired | 114 (29.8) | 23 (45.1) | 91 (27.4) |  |
| BMI (kg/m^2^), Mean (SD) | 24.8 (3.6) | 25.8 (2.9) | 24.6 (3.6) | 0.037 |
| Medical insurance status, n (%) |  |  |  | 0.614 |
| Basic medical insurance | 374 (97.7) | 51 (100.0) | 323 (97.3) |  |
| Government-funded medical insurance | 9 (2.3) | 0 (0.0) | 9 (2.7) |  |
| Smoking history, n (%) |  |  |  | 1.000 |
| Never | 380 (99.2) | 51 (100.0) | 329 (99.1) |  |
| Ever | 3 (0.8) | 0 (0.0) | 3 (0.9) |  |
| ALND affected dominant arm, n (%) |  |  |  | 0.912 |
| None | 198 (51.7) | 26 (51.0) | 172 (51.8) |  |
| Yes | 185 (48.3) | 25 (49.0) | 160 (48.2) |  |
| Affected side, n (%) |  |  |  | 0.623 |
| Left | 200 (52.2) | 25 (49.0) | 175 (52.7) |  |
| Right | 183 (47.8) | 26 (51.0) | 157 (47.3) |  |
| cTNM stage, n (%) |  |  |  | 0.697 |
| I | 3 (0.8) | 0 (0.0) | 3 (0.9) |  |
| II | 322 (84.1) | 45 (88.2) | 277 (83.4) |  |
| III | 58 (15.1) | 6 (11.8) | 52 (15.7) |  |
| pTNM stage, n (%) |  |  |  | 0.112 |
| 0 | 85 (22.2) | 8 (15.7) | 77 (23.2) |  |
| I | 34 (8.9) | 2 (3.9) | 32 (9.6) |  |
| II | 163 (42.6) | 21 (41.2) | 142 (42.8) |  |
| III | 101 (26.4) | 20 (39.2) | 81 (24.4) |  |
| CCI, Median (IQR) | 1.0 (1.0, 2.0) | 2.0 (1.0, 3.0) | 1.0 (0.0, 2.0) | 0.003 |
| ECOG PS, n (%) |  |  |  | 0.133 |
| 0 | 382 (99.7) | 50 (98.0) | 332 (100.0) |  |
| 1 | 1 (0.3) | 1 (2.0) | 0 (0.0) |  |
| Breast surgery, n (%) |  |  |  | 0.644 |
| BCS | 108 (28.2) | 13 (25.5) | 95 (28.6) |  |
| Mastectomy | 275 (71.8) | 38 (74.5) | 237 (71.4) |  |
| No. of nodes removed, Mean (SD) | 21.5 (8.0) | 22.9 (6.4) | 21.3 (8.2) | 0.187 |
| No. of positive nodes, Median (IQR) | 1.0 (0.0, 4.0) | 2.0 (1.0, 8.5) | 1.0 (0.0, 3.0) | 0.011 |
| Chemotherapy |  |  |  | 0.085 |
| None | 42 (11.0) | 10 (19.6) | 32 (9.6) |  |
| Neoadjuvant chemotherapy | 248 (64.8) | 28 (54.9) | 220 (66.3) |  |
| Adjuvant chemotherapy | 93 (24.3) | 13 (25.5) | 80 (24.1) |  |
| Taxane-based chemotherapy, n (%) |  |  |  | 0.328 |
| None | 79 (20.6) | 11 (21.6) | 68 (20.5) |  |
| Neoadjuvant | 206 (53.8) | 23 (45.1) | 183 (55.1) |  |
| Adjuvant | 98 (25.6) | 17 (33.3) | 81 (24.4) |  |
| Radiotherapy, n (%) |  |  |  | 0.031 |
| None | 77 (20.1) | 16 (31.4) | 61 (18.4) |  |
| Yes | 306 (79.9) | 35 (68.6) | 271 (81.6) |  |
| Endocrine therapy, n (%) |  |  |  | 0.126 |
| None | 107 (27.9) | 19 (37.3) | 88 (26.5) |  |
| TAM | 46 (12.0) | 8 (15.7) | 38 (11.4) |  |
| AI | 230 (60.1) | 24 (47.1) | 206 (62.0) |  |

Smoking history was categorized as never (no history of smoking) or ever (former or current smoker).

Abbreviations: SD, standard deviation; IQR, interquartile range; BMI, body mass index; ALND, axillary lymph node dissection; cTNM, clinical tumor-node-metastasis stage; pTNM, pathological tumor-node-metastasis stage; CCI, Charlson Comorbidity Index; ECOG PS, Eastern Cooperative Oncology Group performance status; BCS, breast-conserving surgery; TAM, tamoxifen; AI, aromatase inhibitor.
